# TULIP: a randomised controlled trial of surgical versus non-surgical treatment of lateral compression injuries of the pelvis with complete sacral fractures (LC1) in the non-fragility fracture patient—a feasibility study protocol

**DOI:** 10.1136/bmjopen-2019-036588

**Published:** 2020-02-10

**Authors:** Steven Barnfield, Jenny Ingram, Ruth Halliday, Xavier Griffin, Rosemary Greenwood, Rebecca Kandiyali, Julian Thompson, Joel Glynn, Lucy Beasant, John McArthur, Peter Bates, Mehool Acharya

**Affiliations:** 1 Department of Trauma & Orthopaedics, North Bristol NHS Trust, Southmead Hospital, Bristol, UK; 2 Bristol Medical School, University of Bristol, Bristol, UK; 3 Nuffield Dept of Orthopaedics, Rheumatology and Musculoskeletal Sciences (NDORMS), Kadoorie Centre, John Radcliffe Hospital, Oxford, Oxfordshire, UK; 4 University Hospitals Bristol NHS Foundation Trust, Level 3 Education Centre, Bristol, UK; 5 Department of Anaesthetics, North Bristol NHS Trust, Southmead Hospital, Bristol, UK; 6 Department of Orthopaedics, University Hospitals Coventry and Warwickshire NHS Trust, Coventry, UK; 7 Department of Orthopaedics, Barts Health NHS Trust, London, UK

**Keywords:** trauma management, adult orthopaedics, orthopaedic & trauma surgery

## Abstract

**Introduction:**

Lateral compression type 1 (LC1) pelvic fractures are the most common type of pelvic fracture. The majority of LC1 fractures are considered stable. Fractures where a complete sacral fracture is present increases the degree of potential instability and have the potential to displace over time. Non-operative management of these unstable fractures may involve restricted weight bearing and significant rehabilitation. Frequent monitoring with X-rays is also necessary for displacement of the fracture. Operative stabilisation of these fractures may be appropriate to prevent displacement of the fracture. This may allow patients to mobilise pain-free, quicker.

**Methods and analysis:**

The study is a feasibility study to inform the design of a full definitive randomised controlled trial to guide the most appropriate management of these injuries. Participants will be recruited from major trauma centres and randomly allocated to either operative or non-operative management of their injuries. A variety of outcome instruments, measuring health-related quality of life, functional outcome and pain, will be completed at several time points up to 12 months post injury. Qualitative interviews will be undertaken with participants to explore their views of the treatments under investigation and trial processes.

Eligibility and recruitment to the study will be analysed to inform the feasibility of a definitive trial. Completion rates of the measurement instruments will be assessed, as well as their sensitivity to change and the presence of floor or ceiling effects in this population, to inform the choice of the primary outcome for a definitive trial.

**Ethics and dissemination:**

Ethical approval for the study was given by the South West—Central Bristol NHS Research Ethics Committee on 2nd July 2018 (Ref; 18/SW/0135). The study will be reported in relevant specialist journals and through presentation at specialist conferences.

**Trial registration number:**

ISRCTN10649958

**Strengths and limitations of this study**

- This is the first randomised multicentre study to investigate the treatment of high-energy unstable LC1 fractures.
- We are collecting a range of outcome measures at several time points to identify the most appropriate primary outcome for a definitive study.
- Qualitative interviews will provide valuable insights to identify challenges with recruitment and follow-up and inform the future definitive study design.
- Results of the TULIP feasibility will inform the design and conduct of a future multicentre Randomised Controlled Trial.

## Introduction

### Background

The Trauma Audit and Research Network (TARN) database indicate increasing numbers of pelvic ring fractures. In the financial year 2015/16, TARN recorded 6407 pelvic ring fractures in England and Wales of which half were associated with high-energy trauma. Fractures associated with a side or lateral compression force are the most common; a subgroup of these are called lateral compression type 1 (LC1). LC1 fractures make up approximately 60% of pelvic ring fractures,^1 2^ which equates to approximately 3800 patients a year within England and Wales. A proportion of pelvic fractures are sustained as a result of simple trips or falls and these are generally in the older person where bone quality is frequently poor. Stabilisation of fractures in elderly patients presents technical problems due to the difficulty in achieving adequate fixation in osteoporotic bone. The mortality during index hospital admission associated with LC1 fractures ranges from 5.1% to 8.6%.^1 2^

LC1 fracture patterns are a heterogeneous group of injuries, divided into those involving a complete or an incomplete fracture of the sacrum with or without an injury to the anterior pelvic ring.

The majority of LC1 fractures are considered stable enough to allow rehabilitation without later displacement. Numerous studies have shown complete sacral fractures to be present in 32%–50% of LC1 fractures.^3–5^ The combination of a complete sacral fracture and either unilateral or bilateral pubic rami fractures increases the degree of potential instability. Unstable LC1 fractures of the pelvis have a tendency to displace significantly over time.^6^ Bruce *et al* 5 reported 32% of patients with a complete sacral fracture and unilateral pubic rami fractures, and 68% of patients with a complete sacral fracture and bilateral rami fractures, went on to have significant displacement.

This, potentially unstable, subgroup of LC1 fractures may still be managed non-operatively. Patients would usually be allowed to mobilise as able although they may be advised to restrict the amount of weight they put through the injured side and will require walking aids provided by a physiotherapist. They also require frequent X-rays to monitor for any progression in fracture displacement. Patients with LC1 fractures are reported to spend up to 16 days in hospital following their injury^7^ and require significant rehabilitation following their discharge from acute care.^8^ These injuries can have significant implications for patients. Hoffmann *et al* 9 showed that even at 24 months postinjury, patients had not returned to their preinjury functional abilities. Aprato *et al* 8 found that 60% of the costs following pelvic injury were attributed to health-related work absence.

It may therefore be appropriate to surgically stabilise this subgroup of more severe, potentially unstable, LC1 fractures. This involves the insertion of metalwork to prevent displacement of the fractures. While patients will still require walking aids, their ability to mobilise may be improved. Tosounidis *et al* 7 carried out a non-randomised study comparing surgical versus non-surgical management of LC1 fractures. They found that patients had significantly decreased pain at 72 hours and were able to mobilise, pain-free, quicker following surgery. They also demonstrated a shorter length of stay in patients undergoing surgical fixation. However, Hagen *et al* 10 in a retrospective study looking at patients’ pain, narcotic use and mobility following surgical stabilisation of lateral compression fractures found no significant difference in these parameters between surgically and non-surgically treated groups.

Other advantages of treating these fractures surgically include a lower risk of fracture displacement and avoiding the risks associated with immobility, including chest or urinary tract infection, thrombosis and pressure sores. The disadvantages of treating LC1 fractures surgically are the risk of general anaesthesia, the physiological impact of surgery, the small risk of surgical site infection and of damage to the nerves that supply the bladder, bowel or leg muscles. As well as improving patients’ pain levels and functional abilities, surgery has been shown to provide economic benefits by reducing length of hospital stay and input required from healthcare professionals, which may outweigh the additional costs of surgery.

### Rationale

A survey on the management of LC1 fractures,^11^ although not specific to unstable LC1 fractures, indicated significant variation of practice in managing these fractures and agreement between surgeons was only achieved for one third of case studies.

Both Hagen *et al* 10 and Tosounidis *et al* 7 concluded that a randomised controlled trial of surgical versus nonsurgical management of LC1 fractures was needed. Currently there is no level 1 evidence available to guide clinicians as to the optimum management of these patients.

### Aim and objectives

The overarching aim is to perform a definitive trial to establish whether surgical or non-surgical management of unstable LC1 fractures is most appropriate. The aim of this feasibility study is to allow us to plan a full definitive trial by measuring recruitment, retention and follow-up rates and explore participant and staff views of the trial processes. Study objectives are shown in box 1.

Box 1 **Detailed study objectives**

- To produce a Consolidated Standards of Reporting Trials diagram, reporting screening, recruitment, randomisation compliance and include allocation proportions by centre.
- To confirm the recruitment rates and percentage of eligible patients who agree to take part.
- To collect outcome data at fixed time points post injury to collate the completeness and spread of the data at different time points post injury.
- To identify the outcome measure to be used as the primary outcome on the basis of completeness of data, sensitivity to change over time, the presence of floor or ceiling effects and patient acceptability.
- To develop and refine methods for the collection of resource use data relating to both management pathways.
- To explore patient and staff views of randomisation, treatment and trial processes using qualitative interviews.

## Methods and analysis

### Trial setting

This multicentre trial will take place in 9 NHS Major Trauma Centres (MTC) which specialise in the treatment of pelvic injuries over 33 months. There are 22 MTCs across the UK currently where all patients with unstable pelvic injuries will be referred and assessed.

### Eligibility

All patients over 16 years of age presenting with an LC1 fracture including a complete sacral fracture will be assessed for inclusion in the study. A log of all patients meeting these criteria will be maintained. Patients will be excluded if they meet one of the following criteria:

- Unable to be randomised within 72 hours of having capacity to comprehend the study information following arrival at the major trauma centre.
- Fragility fractures resulting from low-energy trauma (fall from less than standing height).
- Presenting medical condition which precludes surgical intervention.
- Unable to provide informed consent.

### Recruitment

Patients eligible for inclusion in the study will be identified by their surgeon who will make the patient aware of the study and seek their agreement to consider participating. The study will then be fully discussed with the patient by a member of the research team at each site. Patients will be provided with a written information sheet explaining the purpose of the study and the treatments under investigation. They will be allowed sufficient time to consider the information provided and patients who agree to participate in the study will be asked to provide written consent. Patients who decline to participate in the study will be recorded on the screening log together with reasons for declining where provided.

To understand patient perceptions of the recruitment process, all patients that are approached regarding their potential participation in the study will be asked to complete a short questionnaire regardless of whether they consent to participate in the feasibility study. Patients will be asked to complete these questionnaires immediately following confirmation of their decision on participating in the study. Where this is not possible a copy of the questionnaire will be sent in the post by the local research team. Responses to these questionnaires will be confidential and patients will be identified only by their screening ID. The results of this questionnaire will be analysed as an ongoing process to help inform and develop the approach of further patients.

**Figure 1:**
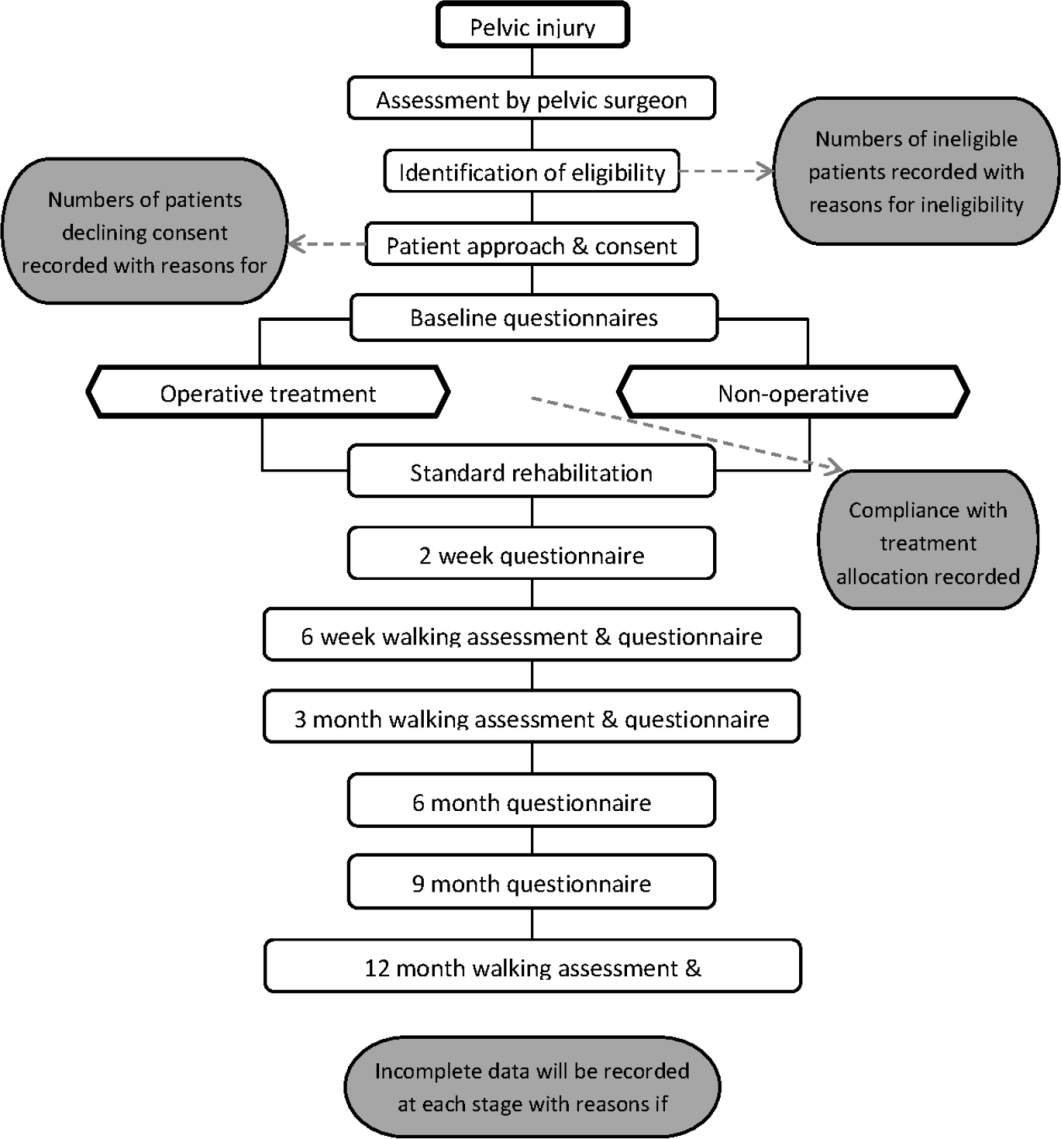
Study flow diagram.

Figure 1 shows the flow of participants through the trial.

### Allocation and blinding

Patients will be randomly allocated to the treatments on a 1:1 basis using a web-based randomisation procedure hosted by Bristol Randomised Trials Collaboration (a registered Clinical Trials Unit) with concealment prior to consent, but no blinding of participants or clinical staff to the allocation of treatment pathway. The trial statistician is responsible for producing the allocation sequence, stratified by recruiting centre and minimised on Injury Severity Score as an indicator of multiple injuries (<16 or>=16).

### Interventions

#### Surgical management

Surgical management will involve fixation of the pelvic fracture by a specialist pelvic surgeon at the earliest opportunity. As surgical fixation of these fractures is performed regularly in all participating centres the method of fixation and choice of implant will be left to the operating surgeon. Postoperative management and rehabilitation will be left to the discretion of the treating surgeon. Details on the surgery and subsequent rehabilitation will be collected as part of the study.

#### Non-surgical management

Non-surgical management will be left to the discretion of the treating surgeon. Any decision on restricted weight-bearing will be left to the treating surgeon. Rehabilitation including Physiotherapy and Occupational Therapy will follow usual practices. Details on the rehabilitation will be collected as part of the study.

### Outcomes

To assess the feasibility of the study design we will assess participant numbers such as recruitment rate, including numbers of patients meeting inclusion criteria and reasons for exclusion or declining where appropriate Compliance rates with allocated treatment and any reasons for not being able to comply. We will also look at follow-up rates, withdrawals, including reasons for withdrawal where appropriate in accordance with the Consolidated Standards of Reporting Trials diagram. We will look at the outcomes measures that are expected to be used in the full trial with particular interest in data completion rates, evidence of sensitivity to change (whether the score change over time) and whether the outcomes have ceiling or floor effects.

The following patient reported outcomes will be tested for use in a definitive study:

### Measures at baseline and follow-up

*Iowa Pelvic Score*: A measure specific to outcomes following pelvic injury.^12^ Shows good construct validity when compared with the physical component of the SF-36. This is also the preferred pelvic specific outcome measure by patients^13^ and the study patient advisory group.

*Oxford Hip Score* 14: A functional score for patients following hip injury and/or surgery. While not pelvic specific, the activities and symptoms included were felt to be relevant by our patient group.

*EQ-5D-5L* 15: A standardised instrument of health status.

*ICECAP-A* 16: A measure of capability for the general adult population for use in economic evaluation. It focuses on well-being in the broader sense, not just health status.

*Brief Pain Inventory* 17: Originally developed to measure pain in patients suffering from cancer. It has since been used in a variety of conditions. It allows patients to rate the severity of their pain as well as its influence on their psychological health and activity

All participants will complete these questionnaires at baseline, 2 and 6 weeks, 3 and 6 months following randomisation. Participants recruited in the first 12 months of the study will also complete questionnaires at 9 and 12 months following randomisation. Baseline data will be collected at recruitment. Participants will be able to complete their follow-up questionnaires in person, when attending an outpatient appointment, online or by post. Standard care for participants with these injuries would be for clinical review in an outpatient clinic at 6 weeks, 3 months and 12 months (see table 1).

**Table 1:** Visit schedule

	Baseline	2 weeks* (+1 week)	6 weeks* (±1 week)	3 months* (±2 weeks)	6 months* (±3 weeks)	9 months* (±3 weeks)	12 months* (±4 weeks)
	Inpatient	Phone/online	Clinic	Clinic	Post/online	Post/online	Clinic
Demographics	✓						
Injury characteristics	✓						
Clinical review	✓	✓†	✓	✓			✓
Surgical details		✓‡					
Rehabilitation		✓	✓	✓	✓	✓	✓
Adverse events	✓	✓	✓	✓	✓	✓	✓
Iowa Pelvic Score	✓§	✓	✓	✓	✓	✓	✓
OHS	✓§	✓	✓	✓	✓	✓	✓
EQ-5D-5L	✓§	✓	✓	✓	✓	✓	✓
ICECAP-A	✓	✓	✓	✓	✓	✓	✓
BPI	✓§	✓	✓	✓	✓	✓	✓
TUAG			✓	✓			✓
Resource use			✓	✓	✓	✓	✓

*From date of randomisation.

†Non-operative group only.

‡Surgical group only.

§Preinjury and postinjury.

BPI, Brief Pain Inventory; EQ-5D-5L, Euroquol - 5 Dimension - 5 level; ICECAP-A, ICEpop Capability measure for Adults; OHS, Oxford Hip Score; TUAG, Timed Up and Go.

At these time points, in addition to the questionnaires, participants will complete a Timed Up and Go assessment.^18^ This is an assessment of a participant’s physical walking ability and involves being timed to stand from a chair, walk a distance of 3 m and return to sit in the chair. Where possible this will be completed by an assessor blinded to the participant’s treatment allocation. Completeness of this assessment will be recorded to inform the appropriateness of its use in a definitive trial.

Data obtained as part of the study will be entered on to a secure password protected online REDCap database.

### Study duration

Recruitment will continue for 18 months. Follow-up for 6 months with 6 months for analysis.

### Economic evaluation

The economic feasibility will focus on data collection to inform the economic evaluation to be done alongside the definitive trial. As well as the EQ-5D-5L and ICECAP-A we will record length of stay in both study arms, along with time spent in theatre and implants used (surgical arm only). Use of specific primary, community and social care services will be assessed by patient reported resource use questionnaires at 6 weeks, 3, 6, 9 and 12 months.

### Qualitative study

To inform the conduct of the definitive trial, we will invite up to 20 consented participants (10 from each treatment arm and across all sites) to take part in a semi-structured telephone interview by the qualitative researcher after they have completed the 6-month follow-up questionnaire. The interviews will explore their experience of the trial, their treatment and recovery, and acceptability of the outcome measures. A purposive sample will be selected to reflect maximum variation in socio-demographics, age and ethnicity. Topic guides for the interviews will be developed from the literature, team discussions and input from the PAG. Ten participating healthcare professionals (surgeons, research nurses and clinical nurse specialists) will be invited to take part in a telephone interview evaluating their experiences of treatment and views of trial processes.

### Safety reporting

Only serious adverse events will be reported for this study comparing two treatments in common clinical practice. A serious adverse event is any untoward medical occurrence that:

- Results in death.
- Is life-threatening.
- Requires inpatient hospitalisation or prolongation of existing hospitalisation.
- Results in persistent or significant disability/incapacity.
- Consists of a congenital anomaly or birth defect.

Serious adverse events which are expected with these injuries are:

- Wound complications/infections.
- Neurovascular injury.
- Thromboembolic events.
- Chest infection.
- Metal work/implant failure/loosening and non/mal-union.

Secondary operations to prevent infection, mal-union, non-union or for symptoms related to the metalwork may also be expected.

Any unexpected serious adverse events will be recorded and reported to the Sponsor and Ethics Committee.

### Sample size

This feasibility study is designed to produce estimates of the parameters required to plan a definitive trial, together with enough data on outcome measures to show whether or not the ceiling effect on the Iowa instrument is likely to be a problem in the definitive trial. If 120 patients are screened as eligible and 40% agree to take part, then this will allow us to estimate the recruitment rate of 40% with a 95% CI of 31%–49% which is within 10% in either direction. Forty complete sets of data should be enough to show when a ceiling effect starts to occur although this will rely on a visual inspection of the data at each time point. If 60 sets of data are collected this will allow greater precision.

### Data analyses

#### Quantitative data analysis

As this is a feasibility trial no formal statistical testing will be carried out. Instead the analysis will focus on reporting data that will be used for planning and for assessing the feasibility of the definitive trial.

Feasibility parameters with 95% CIs will be provided using the exact binomial method. The spread of the data and ceiling effects will be documented for all outcome variables using histograms for single time points and box plots to compare over time. Calculation of the area under the curve over time is the likely primary method of analysis for the definitive trial, and the feasibility analysis will investigate whether this would produce a sufficiently complete data set or whether it would be better to focus on a particular time point. The 95% CI for the effect sizes for all potential outcome measures will be calculated to ensure that a future trial can be planned appropriately.

The future economic evaluation is likely to present results in cost/Quality Adjusted Life Year (QALY) terms reporting within trial and lifetime horizons. The economic feasibility work will focus on establishing the appropriate methods for collecting the outcomes, both costs and utilities, which will be of interest in the future economic evaluation, with analysis therefore limited to assessment of completeness and descriptive statistics.

#### Qualitative data analysis

With informed consent, all interviews will be digitally recorded, transcribed, anonymised and analysed using thematic methods of building codes into themes and sub-themes using the process of constant comparison (facilitated by NVIVO software: QSR International). This aspect is important to understand the acceptability of trial processes, including randomisation, treatment pathways and other outcome questionnaires for the definitive trial.

### Patient and public involvement/patient advisory group

A patient advisory group (PAG) has been involved in the development of the study and advising on study design. The PAG have been particularly involved in the selection of appropriate outcome measures and reviewing patient facing materials including the information sheets. The group will continue to provide advice throughout the study and their advice on any changes which may improve recruitment and the study will be actively sought. A representative of the group will sit on the Trial Steering Committee (TSC) to feedback the advice of the group to the committee. The PAG will also be actively involved in any publication and dissemination of results at the end of the study.

### Dissemination

The findings of the study will be presented locally at each participating site and to the general orthopaedic community at national orthopaedic conferences. The findings will also be submitted for publication in an open access peer-reviewed journal and presented at relevant conferences and research meetings.

## Supplementary Material

Reviewer comments

Author's manuscript
