# Supplementary material for: TULIP: a randomised controlled trial of surgical versus non-surgical treatment of lateral compression injuries of the pelvis with complete sacral fractures (LC1) in the non-fragility fracture patient—a feasibility study protocol
Source: BMJ Open. 2020 Feb 10;10(2):e036588. doi: 10.1136/bmjopen-2019-036588 (PMC7044852; doi:10.1136/bmjopen-2019-036588)
Supplement: Author's manuscript [file bmjopen-2019-036588.draft_revisions.pdf]

BMJ Open

BMJ Open is committed to open peer review. As part of this commitment we make the peer review history of every article we publish publicly available.

When an article is published we post the peer reviewers' comments and the authors' responses online. We also post the versions of the paper that were used during peer review. These are the versions that the peer review comments apply to.

The versions of the paper that follow are the versions that were submitted during the peer review process. They are not the versions of record or the final published versions. They should not be cited or distributed as the published version of this manuscript.

BMJ Open is an open access journal and the full, final, typeset and author-corrected version of record of the manuscript is available on our site with no access controls, subscription charges or pay-per-view fees (http://bmjopen.bmj.com).

If you have any questions on BMJ Open's open peer review process please email info.bmjopen@bmj.com

BMJ Open

TULIP: A Randomised Controlled Trial of Surgical versus Non-Surgical Treatment of Lateral Compression Injuries of the Pelvis with Complete Sacral Fractures (LC1) in the Non-fragility Fracture Patient - A Feasibility Study Protocol.

Journal:	*BMJ Open*
Manuscript ID	bmjopen-2019-036588
Article Type:	Protocol
Date Submitted by the Author:	20-Dec-2019
Complete List of Authors:	Barnfield, Steven; North Bristol NHS Trust,<br>Ingram, Jenny; Bristol University , School of Social & Community Medicine<br>Halliday, Ruth; North Bristol NHS Trust, Trauma Research Team<br>Griffin, Xavier; University of Oxford Nuffield Department of Orthopaedics Rheumatology and Musculoskeletal Sciences, Kadoorie Centre<br>Greenwood, Rosemary; University Hospitals Bristol NHS Foundation Trust, Research and Design Service<br>Kandiyali, Rebecca; University of Bristol School of Social and Community Medicine, Centre for Child and Adolescent Health<br>Thompson, Julian; North Bristol NHS Trust<br>Glynn, Joel; University of Bristol<br>Beasant, Lucy; University of Bristol, School of Social and Community Medicine<br>McArthur, John; University Hospitals Coventry and Warwickshire NHS Trust<br>Bates, Peter; Barts Health NHS Trust<br>Acharya, Mehool; North Bristol NHS Trust, Department of Trauma & Orthopaedics
Keywords:	Trauma management < ORTHOPAEDIC & TRAUMA SURGERY, Adult orthopaedics < ORTHOPAEDIC & TRAUMA SURGERY, Orthopaedic & trauma surgery < SURGERY

SCHOLARONE™
Manuscripts

I, the Submitting Author has the right to grant and does grant on behalf of all authors of the Work (as defined in the below author licence), an exclusive licence and/or a non-exclusive licence for contributions from authors who are: i) UK Crown employees; ii) where BMJ has agreed a CC-BY licence shall apply, and/or iii) in accordance with the terms applicable for US Federal Government officers or employees acting as part of their official duties; on a worldwide, perpetual, irrevocable, royalty-free basis to BMJ Publishing Group Ltd ("BMJ") its licensees and where the relevant Journal is co-owned by BMJ to the co-owners of the Journal, to publish the Work in this journal and any other BMJ products and to exploit all rights, as set out in our licence.

The Submitting Author accepts and understands that any supply made under these terms is made by BMJ to the Submitting Author unless you are acting as an employee on behalf of your employer or a postgraduate student of an affiliated institution which is paying any applicable article publishing charge ("APC") for Open Access articles. Where the Submitting Author wishes to make the Work available on an Open Access basis (and intends to pay the relevant APC), the terms of reuse of such Open Access shall be governed by a Creative Commons licence – details of these licences and which Creative Commons licence will apply to this Work are set out in our licence referred to above.

Other than as permitted in any relevant BMJ Author's Self Archiving Policies, I confirm this Work has not been accepted for publication elsewhere, is not being considered for publication elsewhere and does not duplicate material already published. I confirm all authors consent to publication of this Work and authorise the granting of this licence.

For peer review only

TULIP: A Randomised Controlled Trial of Surgical versus Non-Surgical Treatment of Lateral Compression Injuries of the Pelvis with Complete Sacral Fractures (LC1) in the Non-fragility Fracture Patient - A Feasibility Study Protocol.

Mr Steven Barnfield[1]

Dr Jenny Ingram[2 $]

Miss Ruth Halliday[1]

Associate Professor Xavier Griffin[3]

Mrs Rosemary Greenwood[4]

Dr Rebecca Kandiyali[2]

Dr Julian Thompson[5]

Mr Joel Glynn[2]

Dr Lucy Beasant[2]

Mr John McArthur[6]

Mr Peter Bates[7]

Mr Mehool Acharya[1]

[1] Department of Trauma & Orthopaedics, North Bristol NHS Trust, Southmead Hospital, Bristol, BS10 5NB

[2] University of Bristol, Bristol Medical School,1-5 Whiteladies Road, Bristol, BS8 1NU

[3] Nuffield Dept of Orthopaedics, Rheumatology and Musculoskeletal Sciences (NDORMS), Kadoorie Centre, John Radcliffe Hospital, Headley Way, Oxford, OX3 9DU

[4] University Hospitals Bristol NHS Foundation Trust, Level 3 Education Centre, Upper Maudlin Street, Bristol, BS2 8AE

[5] Department of Anaesthetics, North Bristol NHS Trust, Southmead Hospital, Bristol, BS10 5NB

[6] Department of Orthopaedics, University Hospitals Coventry and Warwickshire NHS Trust, Clifford Bridge Road, Coventry, CV2 2DX

For peer review only

[7] Department of Orthopaedics, Barts Health NHS Trust, Whitechapel Road, London, E1 1BB

[$] Corresponding author,

Email; Jenny.Ingram@bristol.ac.uk, Tel; 0117 428 3095

Protocol Version 4.0, 08/05/2019

Word count: 2968 words

For peer review only

For peer review only - http://bmjopen.bmj.com/site/about/guidelines.xhtml

ABSTRACT

Introduction

Lateral Compression type 1 (LC1) pelvic fractures are the most common type of pelvic fracture (Burgess et al[1], Manson et al[2]). The majority of LC1 fractures are considered stable. Fractures where a complete sacral fracture is present increases the degree of potential instability and have the potential to displace over time. Non-operative management of these unstable fractures may involve restricted weight bearing and significant rehabilitation. Frequent monitoring with x-rays is also necessary for displacement of the fracture. Operative stabilisation of these fractures may be appropriate to prevent displacement of the fracture. This may allow patients to mobilise pain-free, quicker (Tosounidis et al[7]).

Methods and Analysis

The study is a feasibility study to inform the design of a full definitive randomised controlled trial to guide the most appropriate management of these injuries. Participants will be recruited from major trauma centres and randomly allocated to either operative or non-operative management of their injuries. A variety of outcome instruments, measuring health-related quality of life, functional outcome and pain, will be completed at several time points up to 12 months post injury. Qualitative interviews will be undertaken with participants to explore their views of the treatments under investigation and trial processes.

Eligibility and recruitment to the study will be analysed to inform the feasibility of a definitive trial. Completion rates of the measurement instruments will be assessed, as well as their sensitivity to change and the presence of floor or ceiling effects in this population, to inform the choice of the primary outcome for a definitive trial.

Ethics and Dissemination

Ethical approval for the study was given by the South West - Central Bristol NHS Research Ethics Committee on 2nd July 2018 (Ref; 18/SW/0135). The study will be reported in relevant specialist journals and through presentation at specialist conferences.

Trial Registration

ISRCTN; 10649958

For peer review only

STRENGTHS AND LIMITATIONS OF THIS STUDY

- This is the first randomised multicentre study to investigate the treatment of high energy unstable LC1 fractures.
- We are collecting a range of outcome measures at several time points to identify the most appropriate primary outcome for a definitive study.
- Qualitative interviews will provide valuable insights to identify challenges with recruitment and follow-up and inform the future definitive study design.
- Results of the TULIP feasibility will inform the design and conduct of a future multicentre RCT.

For peer review only

INTRODUCTION

Background

The Trauma Audit and Research Network (TARN) database indicate increasing numbers of pelvic ring fractures. In the financial year 2015/16 TARN recorded 6,407 pelvic ring fractures in England and Wales of which half were associated with high energy trauma. Fractures associated with a side or lateral compression force are the most common; a sub-group of these are called lateral compression type 1 (LC1). LC1 fractures make up approximately 60% of pelvic ring fractures[1,2] which equates to approximately 3,800 patients a year within England and Wales. A proportion of pelvic fractures are sustained as a result of simple trips or falls and these are generally in the older person where bone quality is frequently poor. Stabilisation of fractures in elderly patients presents technical problems due to the difficulty in achieving adequate fixation in osteoporotic bone. The mortality during index hospital admission associated with LC1 fractures ranges from 5.1% to 8.6%.[1,2]

LC1 fracture patterns are a heterogeneous group of injuries; divided into those involving a complete or an incomplete fracture of the sacrum with or without an injury to the anterior pelvic ring.

The majority of LC1 fractures are considered stable enough to allow rehabilitation without later displacement. Numerous studies have shown complete sacral fractures to be present in 32-50% of LC1 fractures.[3,4,5] The combination of a complete sacral fracture and either unilateral or bilateral pubic rami fractures increases the degree of potential instability.

For peer review only - http://bmjopen.bmj.com/site/about/guidelines.xhtml

Unstable LC1 fractures of the pelvis have a tendency to displace significantly over time.[6] Bruce et al[5] reported 32% of patients with a complete sacral fracture & unilateral pubic rami fractures, and 68% of patients with a complete sacral fracture & bilateral rami fractures, went on to have significant displacement.

This, potentially unstable, sub-group of LC1 fractures may still be managed non-operatively. Patients would usually be allowed to mobilise as able although they may be advised to restrict the amount of weight they put through the injured side and will require walking aids provided by a physiotherapist. They also require frequent x-rays to monitor for any progression in fracture displacement. Patients with LC1 fractures are reported to spend up to 16 days in hospital following their injury[7] and require significant rehabilitation following their discharge from acute care.[8] These injuries can have significant implications for patients. Hoffmann et al[9] showed that, even at 24 months post injury, patients had not returned to their pre-injury functional abilities. Aprato et al[8] found that 60% of the costs following pelvic injury were attributed to health-related work absence.

It may therefore be appropriate to surgically stabilise this sub-group of more severe, potentially unstable, LC1 fractures. This involves the insertion of metalwork to prevent displacement of the fractures. Whilst patients will still require walking aids, their ability to mobilise may be improved. Tosounidis et al[7] carried out a non-randomised study comparing surgical versus non-surgical management of LC1 fractures. They found that patients had significantly decreased pain at 72 hours and were able to mobilise, pain-free, quicker following surgery. They also demonstrated a shorter length of stay in patients undergoing surgical fixation. However, Hagen et al[10] in a retrospective study looking at patients' pain, narcotic use and mobility following surgical stabilisation of lateral compression fractures found no significant difference in these parameters between surgically and non-surgically treated groups.

Other advantages of treating these fractures surgically include a lower risk of fracture displacement and avoiding the risks associated with immobility, including chest or urinary tract infection, thrombosis and pressure sores. The disadvantages of treating LC1 fractures surgically are the risk of general anaesthesia, the physiological impact of surgery, the small risk of surgical site infection and of damage to the nerves that supply the bladder, bowel or leg muscles. As well as improving patients' pain levels and functional abilities, surgery has been shown to provide economic benefits by reducing length of hospital stay and input required from healthcare professionals, which may outweigh the additional costs of surgery.

For peer review only - http://bmjopen.bmj.com/site/about/guidelines.xhtml

Rationale

A survey on the management of LC1 fractures[11], although not specific to unstable LC1 fractures, indicated significant variation of practice in managing these fractures and agreement between surgeons was only achieved for one third of case studies.

Both Hagen et al[10] and Tosounidis et al[7] concluded that a randomised controlled trial of surgical versus nonsurgical management of LC1 fractures was needed. Currently there is no level 1 evidence available to guide clinicians as to the optimum management of these patients.

Aim and Objectives

The overarching aim is to perform a definitive trial to establish whether surgical or nonsurgical management of unstable LC1 fractures is most appropriate. The aim of this feasibility study is to allow us to plan a full definitive trial by measuring recruitment, retention and follow-up rates and explore participant and staff views of the trial processes. Study objectives are shown in Box 1.

1) To produce a CONSORT (consolidated standards of reporting trials) diagram, reporting screening, recruitment, randomisation compliance and include allocation proportions by centre.

2. To confirm the recruitment rates and percentage of eligible patients who agree to take part.

3. To collect outcome data at fixed time points post injury to collate the completeness and spread of the data at different time points post injury.

4. To identify the outcome measure to be used as the primary outcome on the basis of completeness of data, sensitivity to change over time, the presence of floor or ceiling effects and patient acceptability.

5. To develop and refine methods for the collection of resource use data relating to both management pathways.

6. To explore patient and staff views of randomisation, treatment and trial processes using qualitative interviews.

Box 1: Detailed study objectives

For peer review only - http://bmjopen.bmj.com/site/about/guidelines.xhtml

METHODS AND ANALYSIS

Trial Setting

This multicentre trial will take place in 9 NHS Major Trauma Centres (MTC) which specialise in the treatment of pelvic injuries over 33 months. There are 22 MTCs across the UK currently where all patients with unstable pelvic injuries will be referred and assessed.

Eligibility

All patients over 16 years of age presenting with an LC1 fracture including a complete sacral fracture will be assessed for inclusion in the study. A log of all patients meeting these criteria will be maintained. Patients will be excluded if they meet one of the following criteria:

- Unable to be randomised within 72 hours of having capacity to comprehend the study information following arrival at the major trauma centre.
- Fragility fractures resulting from low-energy trauma (fall from less than standing height).
- Presenting medical condition which precludes surgical intervention.
- Unable to provide informed consent.

Recruitment

Patients eligible for inclusion in the study will be identified by their surgeon who will make the patient aware of the study and seek their agreement to consider participating. The study will then be fully discussed with the patient by a member of the research team at each site. Patients will be provided with a written information sheet explaining the purpose of the study and the treatments under investigation. They will be allowed sufficient time to consider the information provided and patients who agree to participate in the study will be asked to provide written consent. Patients who decline to participate in the study will be recorded on the screening log together with reasons for declining where provided.

To understand patient perceptions of the recruitment process, all patients that are approached regarding their potential participation in the study will be asked to complete a short questionnaire regardless of whether they consent to participate in the feasibility study. Patients will be asked to complete these questionnaires immediately following confirmation of their decision on participating in the study. Where this is not possible a copy of the questionnaire will be sent in the post by the local research team. Responses to these questionnaires will be confidential and patients will be identified only by their screening ID.

For peer review only - http://bmjopen.bmj.com/site/about/guidelines.xhtml

The results of this questionnaire will be analysed as an ongoing process to help inform and develop the approach of further patients.

Figure 1 shows the flow of participants through the trial.

Allocation and Blinding

Patients will be randomly allocated to the treatments on a 1:1 basis using a web-based randomisation procedure hosted by Bristol Randomised Trials Collaboration (a registered CTU) with concealment prior to consent, but no blinding of participants or clinical staff to the allocation of treatment pathway. The trial statistician is responsible for producing the allocation sequence, stratified by recruiting centre and minimised on Injury Severity Score (ISS) as an indicator of multiple injuries (<16 or >=16).

Interventions

Surgical management

Surgical management will involve fixation of the pelvic fracture by a specialist pelvic surgeon at the earliest opportunity. As surgical fixation of these fractures is performed regularly in all participating centres the method of fixation and choice of implant will be left to the operating surgeon. Post-operative management and rehabilitation will be left to the discretion of the treating surgeon. Details on the surgery and subsequent rehabilitation will be collected as part of the study.

Non-surgical management

Non-surgical management will be left to the discretion of the treating surgeon. Any decision on restricted weight-bearing will be left to the treating surgeon. Rehabilitation including Physiotherapy and Occupational Therapy will follow usual practices. Details on the rehabilitation will be collected as part of the study.

Outcomes

To assess the feasibility of the study design we will assess participant numbers such as recruitment rate, including numbers of patients meeting inclusion criteria and reasons for exclusion or declining where appropriate Compliance rates with allocated treatment and any reasons for not being able to comply. We will also look at follow-up rates, withdrawals, including reasons for withdrawal where appropriate in accordance with the CONSORT diagram. We will look at the outcomes measures that are expected to be used in the full trial

For peer review only - http://bmjopen.bmj.com/site/about/guidelines.xhtml

with particular interest in data completion rates, evidence of sensitivity to change (whether the score change over time) and whether the outcomes have ceiling or floor effects.

The following patient reported outcomes will be tested for use in a definitive study:

Measures at baseline and follow-up

Iowa pelvic score - A measure specific to outcomes following pelvic injury (Templeman et al[12]). Shows good construct validity when compared to the physical component of the SF-36. This is also the preferred pelvic specific outcome measure by patients[13] and the study patient advisory group.

Oxford Hip score (Dawson et al[14]) – A functional score for patients following hip injury and/or surgery. Whilst not pelvic specific the activities and symptoms included were felt to be relevant by our patient group.

EQ-5D-5L (Herdman et al[15]) - A standardised instrument of health status.

ICECAP-A (Al-Janabi et al[16]) - A measure of capability for the general adult population for use in economic evaluation. It focuses on wellbeing in the broader sense, not just health status.

Brief Pain Inventory (Cleeland[17]) - Originally developed to measure pain in patients suffering from cancer. It has since been used in a variety of conditions. It allows patients to rate the severity of their pain as well as its influence on their psychological health and activity

All participants will complete these questionnaires at baseline, 2 and 6 weeks, 3 and 6 months following randomisation. Participants recruited in the first 12 months of the study will also complete questionnaires at 9 and 12 months following randomisation. Baseline data will be collected at recruitment. Participants will be able to complete their follow-up questionnaires in person, when attending an outpatient appointment, online or by post. Standard care for participants with these injuries would be for clinical review in an outpatient clinic at 6 weeks, 3 months and 12 months (see figure 1).

At these time points, in addition to the questionnaires, participants will complete a Timed Up and Go assessment (Podsiadlo & Richardson[18]). This is an assessment of a participant's physical walking ability and involves being timed to stand from a chair, walk a distance of 3m and return to sit in the chair. Where possible this will be completed by an assessor blinded to the participant's treatment allocation. Completeness of this assessment will be recorded to inform the appropriateness of its use in a definitive trial.

For peer review only - http://bmjopen.bmj.com/site/about/guidelines.xhtml

Data obtained as part of the study will be entered on to a secure password protected online REDCap database.

Study Duration

Recruitment will continue for 18 months. Follow-up for 6 months with 6 months for analysis.

	Baseline	2 weeks* (+ 1 week)	6 weeks* (+/- 1 week)	3 months* (+/- 2 weeks)	6 months* (+/- 3 weeks)	9 months* (+/- 3 weeks)	12 months* (+/- 4 weeks)
	Inpatient	*Phone/online*	*Clinic*	*Clinic*	*Post/online*	*Post/online*	*Clinic*
**Demographics**	✓						
**Injury characteristics**	✓						
**Clinical review**	✓	✓ [b]	✓	✓			✓
**Surgical details**		✓ [c]					
**Rehabilitation**		✓	✓	✓	✓	✓	✓
**Adverse Events**	✓	✓	✓	✓	✓	✓	✓
**Iowa Pelvic score**	✓ [a]	✓	✓	✓	✓	✓	✓
**OHS**	✓ [a]	✓	✓	✓	✓	✓	✓
**EQ-5D-5L**	✓ [a]	✓	✓	✓	✓	✓	✓
**ICECAP-A**	✓	✓	✓	✓	✓	✓	✓
**BPI**	✓ [a]	✓	✓	✓	✓	✓	✓
**TUAG**			✓	✓			✓
**Resource Use**			✓	✓	✓	✓	✓

[a]pre- & post-injury, [b]non-operative group only, [c]surgical group only, *from date of randomisation

Table 1 - Visit schedule

Economic Evaluation

The economic feasibility will focus on data collection to inform the economic evaluation to be done alongside the definitive trial. As well as the EQ-5D-5L and ICECAP-A we will record length of stay in both study arms, along with time spent in theatre and implants used (surgical arm only). Use of specific primary, community and social care services will be assessed by patient reported resource use questionnaires at 6 weeks, 3, 6, 9 and 12 months.

For peer review only - http://bmjopen.bmj.com/site/about/guidelines.xhtml

Qualitative Study

To inform the conduct of the definitive trial, we will invite up to 20 consented participants (10 from each treatment arm and across all sites) to take part in a semi-structured telephone interview by the qualitative researcher after they have completed the 6 month follow-up questionnaire. The interviews will explore their experience of the trial, their treatment and recovery, and acceptability of the outcome measures. A purposive sample will be selected to reflect maximum variation in socio-demographics, age and ethnicity. Topic guides for the interviews will be developed from the literature, team discussions and input from the PAG. Ten participating health care professionals (surgeons, research nurses and clinical nurse specialists) will be invited to take part in a telephone interview evaluating their experiences of treatment and views of trial processes.

Safety Reporting

Only serious adverse events will be reported for this study comparing two treatments in common clinical practice. A serious adverse event is any untoward medical occurrence that:

- results in death
- is life-threatening
- requires inpatient hospitalisation or prolongation of existing hospitalisation
- results in persistent or significant disability/incapacity
- consists of a congenital anomaly or birth defect

Serious adverse events which are expected with these injuries are:

- Wound complications/infections
- Neurovascular injury
- Thromboembolic events
- Chest infection
- Metal work/implant failure/loosening and non/mal-union

Secondary operations to prevent infection, mal-union, non-union or for symptoms related to the metalwork may also be expected.

Any unexpected serious adverse events will be recorded and reported to the Sponsor and Ethics Committee.

For peer review only

Sample Size

This feasibility study is designed to produce estimates of the parameters required to plan a definitive trial, together with enough data on outcome measures to show whether or not the ceiling effect on the Iowa instrument is likely to be a problem in the definitive trial. If 120 patients are screened as eligible and 40% agree to take part, then this will allow us to estimate the recruitment rate of 40% with a 95% confidence interval of 31% to 49% which is within 10% in either direction. Forty complete sets of data should be enough to show when a ceiling effect starts to occur although this will rely on a visual inspection of the data at each time point. If 60 sets of data are collected this will allow greater precision.

Data Analyses

Quantitative data analysis

As this is a feasibility trial no formal statistical testing will be carried out. Instead the analysis will focus on reporting data that will be used for planning and for assessing the feasibility of the definitive trial.

Feasibility parameters with 95% confidence intervals will be provided using the exact binomial method. The spread of the data and ceiling effects will be documented for all outcome variables using histograms for single time points and box plots to compare over time. Calculation of the area under the curve over time is the likely primary method of analysis for the definitive trial, and the feasibility analysis will investigate whether this would produce a sufficiently complete data set or whether it would be better to focus on a particular time point. The 95% confidence interval for the effect sizes for all potential outcome measures will be calculated to ensure that a future trial can be planned appropriately.

The future economic evaluation is likely to present results in cost/QALY terms reporting within trial and lifetime horizons. The economic feasibility work will focus on establishing the appropriate methods for collecting the outcomes, both costs and utilities, which will be of interest in the future economic evaluation, with analysis therefore limited to assessment of completeness and descriptive statistics.

Qualitative data analysis

With informed consent, all interviews will be digitally recorded, transcribed, anonymised and analysed using thematic methods of building codes into themes and sub-themes using the process of constant comparison (facilitated by NVIVO software: QSR International Pty Ltd).

For peer review only - http://bmjopen.bmj.com/site/about/guidelines.xhtml

This aspect is important to understand the acceptability of trial processes, including randomisation, treatment pathways and other outcome questionnaires for the definitive trial.

Patient and Public Involvement / Patient Advisory Group (PAG)

A patient advisory group (PAG) has been involved in the development of the study and advising on study design. The PAG have been particularly involved in the selection of appropriate outcome measures and reviewing patient facing materials including the information sheets. The group will continue to provide advice throughout the study and their advice on any changes which may improve recruitment and the study will be actively sought. A representative of the group will sit on the TSC to feedback the advice of the group to the committee. The PAG will also be actively involved in any publication and dissemination of results at the end of the study.

ETHICS AND DISSEMINATION

This manuscript is based on Protocol V.4.0 dated 08.05.2019. The study received South West – Central Bristol Research Ethics Committee (REC) approval on 2nd July 2018 and Health Research Authority approval on 4th July 2018. The trial will be conducted in accordance with the protocol, the principles of the Declaration of Helsinki and ICH GCP. Any amendments of the protocol will be submitted to the REC for approval. On request, the study investigators and their institutions will permit trial-related monitoring and audits by the Sponsor and relevant Research Ethics Committee. North Bristol NHS Trust is the nominated sponsor for this study.

A Trial Steering Committee (TSC) has been convened to provide overall supervision of the trial and ensure it is in accordance with the principles of good clinical practice and relevant regulations. The TSC agreed the trial protocol and will agree any protocol amendments. The TSC also provide advice to the investigators on all aspects of the trial including aspects of safety and monitoring of serious adverse events.

Dissemination

The findings of the study will be presented locally at each participating site and to the general orthopaedic community at national orthopaedic conferences. The findings will also

For peer review only - http://bmjopen.bmj.com/site/about/guidelines.xhtml

be submitted for publication in an open access peer-reviewed journal and presented at relevant conferences and research meetings.

For peer review only

For peer review only - http://bmjopen.bmj.com/site/about/guidelines.xhtml

REFERENCES

1. Burgess, A., Eastridge, B., Young, J., Ellison, T., Ellison, P., Poka, A., Bathon, G., Brumback, R. (1990) Pelvic ring disruptions: effective classification system and treatment protocols. *Journal of Trauma,* 30(7), pp848–856.
2. Manson, T., O'Toole, R., Whitney, A., Duggan, B., Sciadini, M., Nascone, J. (2010) Young-Burgess classification of pelvic ring fractures: does it predict mortality, transfusion requirements, and non-orthopaedic injuries? *Journal of Orthopaedic Trauma,* 24(10), pp603–609.
3. LeFaivre, K., Padalecki, J., Starr, A. (2009) What constitutes a Young and Burgess lateral compression-I (OTA 61-B2) pelvic ring disruption? A description of computed tomography-based fracture anatomy and associated injuries. *Journal of Orthopaedic Trauma,* 23, pp16–21.
4. Weaver, M., Bruinsma, W., Toney, E., Dafford, E., Vrahas, M. (2012) What are the patterns of injury and displacement seen in lateral compression pelvic fractures? *Clinical Orthopaedics and Related Research,* 470(8), pp2104-2110.
5. Bruce, B., Reilly, M., Sims, S. (2011) OTA Highlight paper predicting future displacement of nonoperatively managed Lateral Compression Sacral fractures: Can it be done? *Journal of Orthopaedic Trauma,* 25(9), pp523-527.
6. Sagi, H., Coniglione, F., Stanford, J. (2011) Examination under anesthetic for occult pelvic ring instability. *Journal of Orthopaedic Trauma,* 25, pp529–536.
7. Tosounidis, T., Kanakaris, N., Nikolaou, V., Tan, B., Giannoudis, P (2012) Assessment of lateral compression type 1 pelvic ring injuries by intraoperative manipulation: which fracture pattern is unstable? *International Orthopaedics,* 36, pp2553-2558.
8. Aprato, A., Joeris, A., Tosto, F., Kalampoki, V., Stucchi, A., Masse, A. (2016) Direct and indirect costs of surgically treated pelvic fractures. *Archives of Orthopaedic Trauma and Surgery,* 136, pp325-330.
9. Hoffmann, M., Jones, C., Sietsma, D. (2012) Persistent impairment after surgically treated lateral compression pelvic injury. *Clinical Orthopaedics and Related Research,* 470, pp2161-2172.
10. Hagen, J., Castillo, R., Dubina, A., Gaski, G., Manson, T. & O'Toole, R. (2016) Does surgical stabilization of lateral compression-type pelvic ring fractures decrease patients' pain, reduce narcotic use and improve mobilization? *Clinical Orthopaedics and Related Research,* 474(6), pp1422-1429.
11. Beckmann, J., Presson, A., Curtis, S., Haller, J., Stuart, A., Higgins, T., Kubiak, E. (2014) Operative agreement on lateral compression-1 pelvis fractures: A survey of 111 OTA members. *Journal of Orthopaedic Trauma,* 28(12), pp681-685.

For peer review only - http://bmjopen.bmj.com/site/about/guidelines.xhtml

12. Templeman, D., Goulet, J., Duwelius, P., Olson, S., Davidson, M. (1996) Internal fixation of displaced fractures of the sacrum. *Clinical Orthopaedics and Related Research,* 329, pp180-185.
13. LeFaivre, K., Slobogean, G., Ngai, J., Broekhuyse, H., O'Brien, P. (2014) What outcomes are important for patients after pelvic trauma? Subjective responses and psychometric analysis of three published pelvic-specific outcome instruments. *Journal of Orthopaedic Trauma,* 28(1), pp23-27.
14. Dawson, J., Fitzpatrick, R., Carr, A., Murray, D. (1996) Questionnaire on the perceptions of patients about total hip replacement. *Journal of Bone and Joint Surgery,* 78-B(2), pp185-190.
15. Herdman, M., Gudex, C., Lloyd, A., Janssen, M., Kind, P., Parkin, D., Bonsel, G., Badia, X. (2011) Development and preliminary testing of the new five-level version of EQ-5D (EQ-5D-5L) *Quality of Life Research,* 20(10), pp1727-1736.
16. Al-Janabi H, Flynn T, Coast J. (2012) Development of a self-report measure of capability wellbeing for adults: the ICECAP-A. *Quality of Life Research,* 21, pp167-176
17. Cleeland, C. (1989) Measurement of pain by subjective report. In: Chapman, C., Loeser, J. (eds) Advances in Pain Research and Therapy, Volume 12: Issues in Pain Measurement. New York: Raven Press, pp391-403.
18. Podsiadlo, D., Richardson, S. (1991) The timed "Up & Go": a test of basic functional mobility for frail elderly persons. *Journal of the American Geriatric Society,* 39(2), pp142-148.

For peer review only

For peer review only - http://bmjopen.bmj.com/site/about/guidelines.xhtml

Acknowledgements The authors would like to thank the patient representatives who contribute to the trial management and steering groups.

Author Contributions: SB wrote the manuscript. MA, SB, RH, JI, RG, RK, XG, JM, PB, JT are all key protocol contributors providing expertise on specific aspects. MA is the chief investigator. All authors have read, contributed amendments to and approved the final manuscript.

Funding: This work is funded by the National Institute for Health Research (NIHR) Research for Patient Benefit research programme grant number PB-PG-0816-20013.

This work is supported by the National Institute for Health Research Oxford Biomedical Research Centre

Disclaimer: This paper presents independent research funded by the National Institute for Health Research (NIHR) under its Research for Patient Benefit (RfPB) Programme (Grant Reference Number PB-PG-0816-20013). The views expressed are those of the author(s) and not necessarily those of the NIHR or the Department of Health and Social Care.

Competing interests: Xavier Griffin is funded by a National Institute for Health Research Clinician Scientist Grant. Further funding from industry and charitable grants are and have been made available to his institution. All decisions relating to the design, conduct, analysis, write-up and publication of this research are independent of these funders. He has ongoing expert consultancy with several companies; none involve the development of any implant for use in pelvic fracture care.

No other authors have competing interests to declare.

Provenance and peer review: Not commissioned; externally peer reviewed.

Data sharing statement: The results of this feasibility study will inform the planning of a definitive randomised controlled trial, including whether it can be conducted, by assessing rates of recruitment, retention and data collection. Data will be made available upon reasonable request.

Trial status: Ongoing data collection.

Trial sponsor: North Bristol NHS Trust, Bristol BS10 5NB: researchsponsor@nbt.nhs.uk

For peer review only

For peer review only

For peer review only - http://bmjopen.bmj.com/site/about/guidelines.xhtml

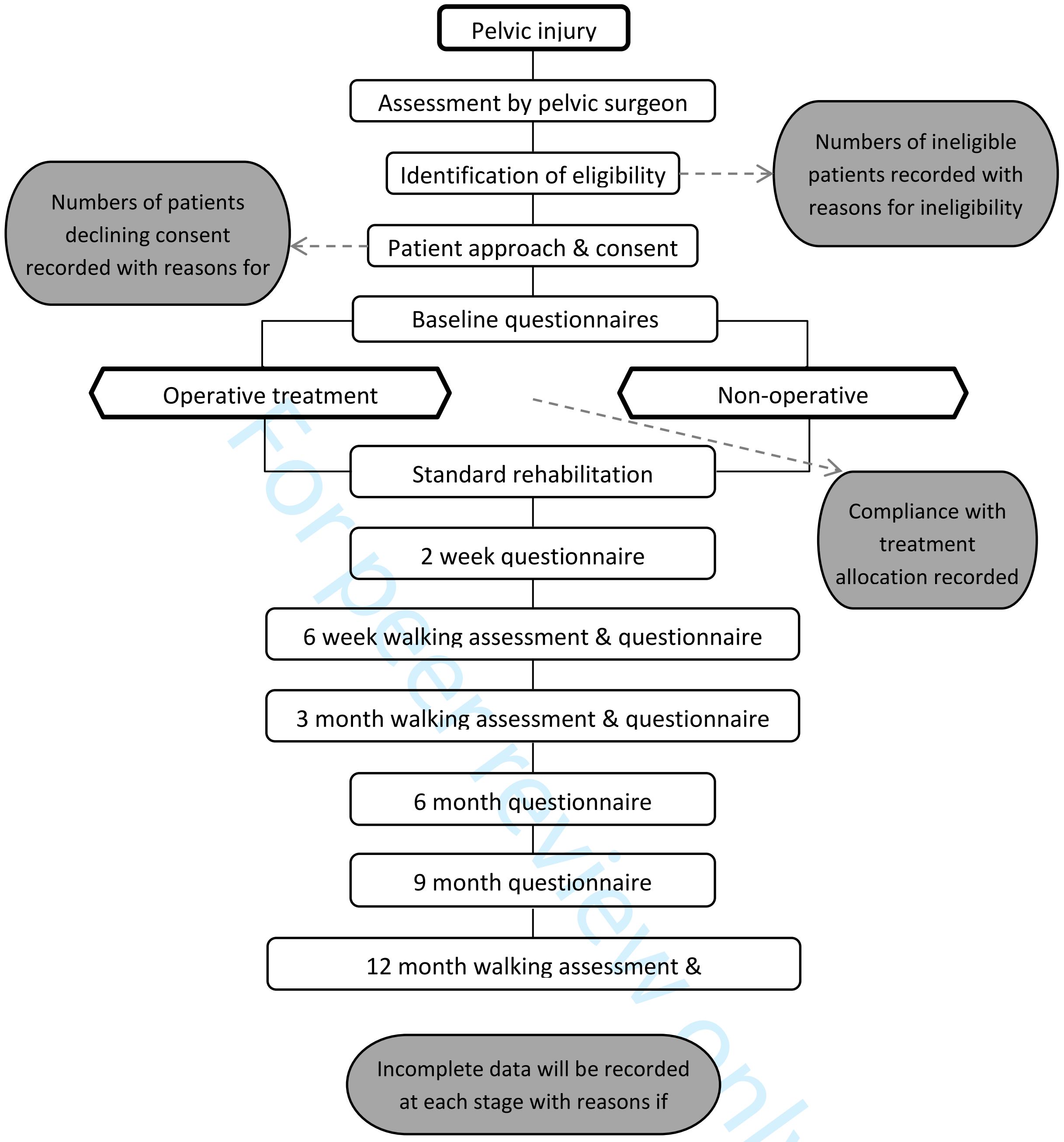

Figure 1 Study flow diagram

For peer review only

SPIRIT 2013 Checklist: Recommended items to address in a clinical trial protocol and related documents*

Section/item	Item No	Description	Addressed on page number
**Administrative information**			
Title	1	Descriptive title identifying the study design, population, interventions, and, if applicable, trial acronym	_____1_______
Trial registration	2a	Trial identifier and registry name. If not yet registered, name of intended registry	_____3_______
	2b	All items from the World Health Organization Trial Registration Data Set	_____n/a_____
Protocol version	3	Date and version identifier	_____13_______
Funding	4	Sources and types of financial, material, and other support	_____17_______
Roles and responsibilities	5a	Names, affiliations, and roles of protocol contributors	_____1________
	5b	Name and contact information for the trial sponsor	_____17_______
	5c	Role of study sponsor and funders, if any, in study design; collection, management, analysis, and interpretation of data; writing of the report; and the decision to submit the report for publication, including whether they will have ultimate authority over any of these activities	_____n/a______
	5d	Composition, roles, and responsibilities of the coordinating centre, steering committee, endpoint adjudication committee, data management team, and other individuals or groups overseeing the trial, if applicable (see Item 21a for data monitoring committee)	_______n/a_____

For peer review only

Introduction

Background and rationale	6a	Description of research question and justification for undertaking the trial, including summary of relevant studies (published and unpublished) examining benefits and harms for each intervention	____4_______
	6b	Explanation for choice of comparators	_____6______
Objectives	7	Specific objectives or hypotheses	_____6______
Trial design	8	Description of trial design including type of trial (eg, parallel group, crossover, factorial, single group), allocation ratio, and framework (eg, superiority, equivalence, noninferiority, exploratory)	_______6______

Methods: Participants, interventions, and outcomes

Study setting	9	Description of study settings (eg, community clinic, academic hospital) and list of countries where data will be collected. Reference to where list of study sites can be obtained	_____7______
Eligibility criteria	10	Inclusion and exclusion criteria for participants. If applicable, eligibility criteria for study centres and individuals who will perform the interventions (eg, surgeons, psychotherapists)	_____7_______
Interventions	11a	Interventions for each group with sufficient detail to allow replication, including how and when they will be administered	_____8______
	11b	Criteria for discontinuing or modifying allocated interventions for a given trial participant (eg, drug dose change in response to harms, participant request, or improving/worsening disease)	_____n/a_____
	11c	Strategies to improve adherence to intervention protocols, and any procedures for monitoring adherence (eg, drug tablet return, laboratory tests)	_____n/a_____
	11d	Relevant concomitant care and interventions that are permitted or prohibited during the trial	_____8______
Outcomes	12	Primary, secondary, and other outcomes, including the specific measurement variable (eg, systolic blood pressure), analysis metric (eg, change from baseline, final value, time to event), method of aggregation (eg, median, proportion), and time point for each outcome. Explanation of the clinical relevance of chosen efficacy and harm outcomes is strongly recommended	_____9______
Participant timeline	13	Time schedule of enrolment, interventions (including any run-ins and washouts), assessments, and visits for participants. A schematic diagram is highly recommended (see Figure)	_____10_____

For peer review only - http://bmjopen.bmj.com/site/about/guidelines.xhtml

Sample size	14	Estimated number of participants needed to achieve study objectives and how it was determined, including clinical and statistical assumptions supporting any sample size calculations	_____12_____
Recruitment	15	Strategies for achieving adequate participant enrolment to reach target sample size	_____7______

Methods: Assignment of interventions (for controlled trials)

Allocation:

Sequence generation	16a	Method of generating the allocation sequence (eg, computer-generated random numbers), and list of any factors for stratification. To reduce predictability of a random sequence, details of any planned restriction (eg, blocking) should be provided in a separate document that is unavailable to those who enrol participants or assign interventions	______8______
Allocation concealment mechanism	16b	Mechanism of implementing the allocation sequence (eg, central telephone; sequentially numbered, opaque, sealed envelopes), describing any steps to conceal the sequence until interventions are assigned	______8______
Implementation	16c	Who will generate the allocation sequence, who will enrol participants, and who will assign participants to interventions	______8______
Blinding (masking)	17a	Who will be blinded after assignment to interventions (eg, trial participants, care providers, outcome assessors, data analysts), and how	______8_____
	17b	If blinded, circumstances under which unblinding is permissible, and procedure for revealing a participant's allocated intervention during the trial	_____n/a_____

Methods: Data collection, management, and analysis

Data collection methods	18a	Plans for assessment and collection of outcome, baseline, and other trial data, including any related processes to promote data quality (eg, duplicate measurements, training of assessors) and a description of study instruments (eg, questionnaires, laboratory tests) along with their reliability and validity, if known. Reference to where data collection forms can be found, if not in the protocol	_____9______
	18b	Plans to promote participant retention and complete follow-up, including list of any outcome data to be collected for participants who discontinue or deviate from intervention protocols	______n/a______

For peer review only - http://bmjopen.bmj.com/site/about/guidelines.xhtml

Data management	19	Plans for data entry, coding, security, and storage, including any related processes to promote data quality (eg, double data entry; range checks for data values). Reference to where details of data management procedures can be found, if not in the protocol	______10______
Statistical methods	20a	Statistical methods for analysing primary and secondary outcomes. Reference to where other details of the statistical analysis plan can be found, if not in the protocol	______12______
	20b	Methods for any additional analyses (eg, subgroup and adjusted analyses)	______12______
	20c	Definition of analysis population relating to protocol non-adherence (eg, as randomised analysis), and any statistical methods to handle missing data (eg, multiple imputation)	______n/a_____
**Methods: Monitoring**			
Data monitoring	21a	Composition of data monitoring committee (DMC); summary of its role and reporting structure; statement of whether it is independent from the sponsor and competing interests; and reference to where further details about its charter can be found, if not in the protocol. Alternatively, an explanation of why a DMC is not needed	______n/a______
	21b	Description of any interim analyses and stopping guidelines, including who will have access to these interim results and make the final decision to terminate the trial	_____n/a_____
Harms	22	Plans for collecting, assessing, reporting, and managing solicited and spontaneously reported adverse events and other unintended effects of trial interventions or trial conduct	_____11______
Auditing	23	Frequency and procedures for auditing trial conduct, if any, and whether the process will be independent from investigators and the sponsor	_____n/a_____
**Ethics and dissemination**			
Research ethics approval	24	Plans for seeking research ethics committee/institutional review board (REC/IRB) approval	_____13_____
Protocol amendments	25	Plans for communicating important protocol modifications (eg, changes to eligibility criteria, outcomes, analyses) to relevant parties (eg, investigators, REC/IRBs, trial participants, trial registries, journals, regulators)	_____13_____

For peer review only - http://bmjopen.bmj.com/site/about/guidelines.xhtml

Consent or assent	26a	Who will obtain informed consent or assent from potential trial participants or authorised surrogates, and how (see Item 32)	_____7______
	26b	Additional consent provisions for collection and use of participant data and biological specimens in ancillary studies, if applicable	____n/a______
Confidentiality	27	How personal information about potential and enrolled participants will be collected, shared, and maintained in order to protect confidentiality before, during, and after the trial	_____n/a_____
Declaration of interests	28	Financial and other competing interests for principal investigators for the overall trial and each study site	_____17______
Access to data	29	Statement of who will have access to the final trial dataset, and disclosure of contractual agreements that limit such access for investigators	_____n/a_____
Ancillary and post-trial care	30	Provisions, if any, for ancillary and post-trial care, and for compensation to those who suffer harm from trial participation	_____n/a_____
Dissemination policy	31a	Plans for investigators and sponsor to communicate trial results to participants, healthcare professionals, the public, and other relevant groups (eg, via publication, reporting in results databases, or other data sharing arrangements), including any publication restrictions	_____13______
	31b	Authorship eligibility guidelines and any intended use of professional writers	_____n/a______
	31c	Plans, if any, for granting public access to the full protocol, participant-level dataset, and statistical code	_____n/a______
**Appendices**			
Informed consent materials	32	Model consent form and other related documentation given to participants and authorised surrogates	_____n/a______
Biological specimens	33	Plans for collection, laboratory evaluation, and storage of biological specimens for genetic or molecular analysis in the current trial and for future use in ancillary studies, if applicable	_____n/a______

*It is strongly recommended that this checklist be read in conjunction with the SPIRIT 2013 Explanation & Elaboration for important clarification on the items. Amendments to the protocol should be tracked and dated. The SPIRIT checklist is copyrighted by the SPIRIT Group under the Creative Commons "Attribution-NonCommercial-NoDerivs 3.0 Unported" license.

BMJ Open

TULIP: A Randomised Controlled Trial of Surgical versus Non-Surgical Treatment of Lateral Compression Injuries of the Pelvis with Complete Sacral Fractures (LC1) in the Non-fragility Fracture Patient - A Feasibility Study Protocol.

Journal:	*BMJ Open*
Manuscript ID	bmjopen-2019-036588.R1
Article Type:	Protocol
Date Submitted by the Author:	15-Jan-2020

Complete List of Authors:	Barnfield, Steven; North Bristol NHS Trust,<br>Ingram, Jenny; Bristol University , School of Social & Community Medicine<br>Halliday, Ruth; North Bristol NHS Trust, Trauma Research Team<br>Griffin, Xavier; University of Oxford Nuffield Department of Orthopaedics Rheumatology and Musculoskeletal Sciences, Kadoorie Centre<br>Greenwood, Rosemary; University Hospitals Bristol NHS Foundation Trust, Research and Design Service<br>Kandiyali, Rebecca; University of Bristol School of Social and Community Medicine, Centre for Child and Adolescent Health<br>Thompson, Julian; North Bristol NHS Trust<br>Glynn, Joel; University of Bristol<br>Beasant, Lucy; University of Bristol, School of Social and Community Medicine<br>McArthur, John; University Hospitals Coventry and Warwickshire NHS Trust<br>Bates, Peter; Barts Health NHS Trust<br>Acharya, Mehool; North Bristol NHS Trust, Department of Trauma & Orthopaedics

**Primary Subject Heading**:	Surgery
Secondary Subject Heading:	Emergency medicine
Keywords:	Trauma management < ORTHOPAEDIC & TRAUMA SURGERY, Adult orthopaedics < ORTHOPAEDIC & TRAUMA SURGERY, Orthopaedic & trauma surgery < SURGERY

SCHOLARONE™ Manuscripts

I, the Submitting Author has the right to grant and does grant on behalf of all authors of the Work (as defined in the below author licence), an exclusive licence and/or a non-exclusive licence for contributions from authors who are: i) UK Crown employees; ii) where BMJ has agreed a CC-BY licence shall apply, and/or iii) in accordance with the terms applicable for US Federal Government officers or employees acting as part of their official duties; on a worldwide, perpetual, irrevocable, royalty-free basis to BMJ Publishing Group Ltd ("BMJ") its licensees and where the relevant Journal is co-owned by BMJ to the co-owners of the Journal, to publish the Work in this journal and any other BMJ products and to exploit all rights, as set out in our licence.

The Submitting Author accepts and understands that any supply made under these terms is made by BMJ to the Submitting Author unless you are acting as an employee on behalf of your employer or a postgraduate student of an affiliated institution which is paying any applicable article publishing charge ("APC") for Open Access articles. Where the Submitting Author wishes to make the Work available on an Open Access basis (and intends to pay the relevant APC), the terms of reuse of such Open Access shall be governed by a Creative Commons licence – details of these licences and which Creative Commons licence will apply to this Work are set out in our licence referred to above.

Other than as permitted in any relevant BMJ Author's Self Archiving Policies, I confirm this Work has not been accepted for publication elsewhere, is not being considered for publication elsewhere and does not duplicate material already published. I confirm all authors consent to publication of this Work and authorise the granting of this licence.

For peer review only

TULIP: A Randomised Controlled Trial of Surgical versus Non-Surgical Treatment of Lateral Compression Injuries of the Pelvis with Complete Sacral Fractures (LC1) in the Non-fragility Fracture Patient - A Feasibility Study Protocol.

Mr Steven Barnfield[1]

Dr Jenny Ingram[2 $]

Miss Ruth Halliday[1]

Associate Professor Xavier Griffin[3]

Mrs Rosemary Greenwood[4]

Dr Rebecca Kandiyali[2]

Dr Julian Thompson[5]

Mr Joel Glynn[2]

Dr Lucy Beasant[2]

Mr John McArthur[6]

Mr Peter Bates[7]

Mr Mehool Acharya[1]

[1] Department of Trauma & Orthopaedics, North Bristol NHS Trust, Southmead Hospital, Bristol, BS10 5NB

[2] University of Bristol, Bristol Medical School,1-5 Whiteladies Road, Bristol, BS8 1NU

[3] Nuffield Dept of Orthopaedics, Rheumatology and Musculoskeletal Sciences (NDORMS), Kadoorie Centre, John Radcliffe Hospital, Headley Way, Oxford, OX3 9DU

[4] University Hospitals Bristol NHS Foundation Trust, Level 3 Education Centre, Upper Maudlin Street, Bristol, BS2 8AE

[5] Department of Anaesthetics, North Bristol NHS Trust, Southmead Hospital, Bristol, BS10 5NB

[6] Department of Orthopaedics, University Hospitals Coventry and Warwickshire NHS Trust, Clifford Bridge Road, Coventry, CV2 2DX

For peer review only

[7] Department of Orthopaedics, Barts Health NHS Trust, Whitechapel Road, London, E1 1BB

[$] Corresponding author,

Email; Jenny.Ingram@bristol.ac.uk, Tel; 0117 428 3095

Protocol Version 4.0, 08/05/2019

Word count: 2968 words

For peer review only

For peer review only - http://bmjopen.bmj.com/site/about/guidelines.xhtml

ABSTRACT

Introduction

Lateral Compression type 1 (LC1) pelvic fractures are the most common type of pelvic fracture (Burgess et al[1], Manson et al[2]). The majority of LC1 fractures are considered stable. Fractures where a complete sacral fracture is present increases the degree of potential instability and have the potential to displace over time. Non-operative management of these unstable fractures may involve restricted weight bearing and significant rehabilitation. Frequent monitoring with x-rays is also necessary for displacement of the fracture. Operative stabilisation of these fractures may be appropriate to prevent displacement of the fracture. This may allow patients to mobilise pain-free, quicker (Tosounidis et al[7]).

Methods and Analysis

The study is a feasibility study to inform the design of a full definitive randomised controlled trial to guide the most appropriate management of these injuries. Participants will be recruited from major trauma centres and randomly allocated to either operative or non-operative management of their injuries. A variety of outcome instruments, measuring health-related quality of life, functional outcome and pain, will be completed at several time points up to 12 months post injury. Qualitative interviews will be undertaken with participants to explore their views of the treatments under investigation and trial processes.

Eligibility and recruitment to the study will be analysed to inform the feasibility of a definitive trial. Completion rates of the measurement instruments will be assessed, as well as their sensitivity to change and the presence of floor or ceiling effects in this population, to inform the choice of the primary outcome for a definitive trial.

Ethics and Dissemination

Ethical approval for the study was given by the South West - Central Bristol NHS Research Ethics Committee on 2nd July 2018 (Ref; 18/SW/0135). The study will be reported in relevant specialist journals and through presentation at specialist conferences.

Trial Registration

ISRCTN; 10649958

For peer review only - http://bmjopen.bmj.com/site/about/guidelines.xhtml

STRENGTHS AND LIMITATIONS OF THIS STUDY

- This is the first randomised multicentre study to investigate the treatment of high energy unstable LC1 fractures.
- We are collecting a range of outcome measures at several time points to identify the most appropriate primary outcome for a definitive study.
- Qualitative interviews will provide valuable insights to identify challenges with recruitment and follow-up and inform the future definitive study design.
- Results of the TULIP feasibility will inform the design and conduct of a future multicentre RCT.

For peer review only

INTRODUCTION

Background

The Trauma Audit and Research Network (TARN) database indicate increasing numbers of pelvic ring fractures. In the financial year 2015/16 TARN recorded 6,407 pelvic ring fractures in England and Wales of which half were associated with high energy trauma. Fractures associated with a side or lateral compression force are the most common; a sub-group of these are called lateral compression type 1 (LC1). LC1 fractures make up approximately 60% of pelvic ring fractures[1,2] which equates to approximately 3,800 patients a year within England and Wales. A proportion of pelvic fractures are sustained as a result of simple trips or falls and these are generally in the older person where bone quality is frequently poor. Stabilisation of fractures in elderly patients presents technical problems due to the difficulty in achieving adequate fixation in osteoporotic bone. The mortality during index hospital admission associated with LC1 fractures ranges from 5.1% to 8.6%.[1,2]

LC1 fracture patterns are a heterogeneous group of injuries; divided into those involving a complete or an incomplete fracture of the sacrum with or without an injury to the anterior pelvic ring.

The majority of LC1 fractures are considered stable enough to allow rehabilitation without later displacement. Numerous studies have shown complete sacral fractures to be present in 32-50% of LC1 fractures.[3,4,5] The combination of a complete sacral fracture and either unilateral or bilateral pubic rami fractures increases the degree of potential instability.

For peer review only - http://bmjopen.bmj.com/site/about/guidelines.xhtml

Unstable LC1 fractures of the pelvis have a tendency to displace significantly over time.[6] Bruce et al[5] reported 32% of patients with a complete sacral fracture & unilateral pubic rami fractures, and 68% of patients with a complete sacral fracture & bilateral rami fractures, went on to have significant displacement.

This, potentially unstable, sub-group of LC1 fractures may still be managed non-operatively. Patients would usually be allowed to mobilise as able although they may be advised to restrict the amount of weight they put through the injured side and will require walking aids provided by a physiotherapist. They also require frequent x-rays to monitor for any progression in fracture displacement. Patients with LC1 fractures are reported to spend up to 16 days in hospital following their injury[7] and require significant rehabilitation following their discharge from acute care.[8] These injuries can have significant implications for patients. Hoffmann et al[9] showed that, even at 24 months post injury, patients had not returned to their pre-injury functional abilities. Aprato et al[8] found that 60% of the costs following pelvic injury were attributed to health-related work absence.

It may therefore be appropriate to surgically stabilise this sub-group of more severe, potentially unstable, LC1 fractures. This involves the insertion of metalwork to prevent displacement of the fractures. Whilst patients will still require walking aids, their ability to mobilise may be improved. Tosounidis et al[7] carried out a non-randomised study comparing surgical versus non-surgical management of LC1 fractures. They found that patients had significantly decreased pain at 72 hours and were able to mobilise, pain-free, quicker following surgery. They also demonstrated a shorter length of stay in patients undergoing surgical fixation. However, Hagen et al[10] in a retrospective study looking at patients' pain, narcotic use and mobility following surgical stabilisation of lateral compression fractures found no significant difference in these parameters between surgically and non-surgically treated groups.

Other advantages of treating these fractures surgically include a lower risk of fracture displacement and avoiding the risks associated with immobility, including chest or urinary tract infection, thrombosis and pressure sores. The disadvantages of treating LC1 fractures surgically are the risk of general anaesthesia, the physiological impact of surgery, the small risk of surgical site infection and of damage to the nerves that supply the bladder, bowel or leg muscles. As well as improving patients' pain levels and functional abilities, surgery has been shown to provide economic benefits by reducing length of hospital stay and input required from healthcare professionals, which may outweigh the additional costs of surgery.

For peer review only - http://bmjopen.bmj.com/site/about/guidelines.xhtml

Rationale

A survey on the management of LC1 fractures[11], although not specific to unstable LC1 fractures, indicated significant variation of practice in managing these fractures and agreement between surgeons was only achieved for one third of case studies.

Both Hagen et al[10] and Tosounidis et al[7] concluded that a randomised controlled trial of surgical versus nonsurgical management of LC1 fractures was needed. Currently there is no level 1 evidence available to guide clinicians as to the optimum management of these patients.

Aim and Objectives

The overarching aim is to perform a definitive trial to establish whether surgical or nonsurgical management of unstable LC1 fractures is most appropriate. The aim of this feasibility study is to allow us to plan a full definitive trial by measuring recruitment, retention and follow-up rates and explore participant and staff views of the trial processes. Study objectives are shown in Box 1.

1) To produce a CONSORT (consolidated standards of reporting trials) diagram, reporting screening, recruitment, randomisation compliance and include allocation proportions by centre.

2. To confirm the recruitment rates and percentage of eligible patients who agree to take part.

3. To collect outcome data at fixed time points post injury to collate the completeness and spread of the data at different time points post injury.

4. To identify the outcome measure to be used as the primary outcome on the basis of completeness of data, sensitivity to change over time, the presence of floor or ceiling effects and patient acceptability.

5. To develop and refine methods for the collection of resource use data relating to both management pathways.

6. To explore patient and staff views of randomisation, treatment and trial processes using qualitative interviews.

Box 1: Detailed study objectives

For peer review only - http://bmjopen.bmj.com/site/about/guidelines.xhtml

METHODS AND ANALYSIS

Trial Setting

This multicentre trial will take place in 9 NHS Major Trauma Centres (MTC) which specialise in the treatment of pelvic injuries over 33 months. There are 22 MTCs across the UK currently where all patients with unstable pelvic injuries will be referred and assessed.

Eligibility

All patients over 16 years of age presenting with an LC1 fracture including a complete sacral fracture will be assessed for inclusion in the study. A log of all patients meeting these criteria will be maintained. Patients will be excluded if they meet one of the following criteria:

- Unable to be randomised within 72 hours of having capacity to comprehend the study information following arrival at the major trauma centre.
- Fragility fractures resulting from low-energy trauma (fall from less than standing height).
- Presenting medical condition which precludes surgical intervention.
- Unable to provide informed consent.

Recruitment

Patients eligible for inclusion in the study will be identified by their surgeon who will make the patient aware of the study and seek their agreement to consider participating. The study will then be fully discussed with the patient by a member of the research team at each site. Patients will be provided with a written information sheet explaining the purpose of the study and the treatments under investigation. They will be allowed sufficient time to consider the information provided and patients who agree to participate in the study will be asked to provide written consent. Patients who decline to participate in the study will be recorded on the screening log together with reasons for declining where provided.

To understand patient perceptions of the recruitment process, all patients that are approached regarding their potential participation in the study will be asked to complete a short questionnaire regardless of whether they consent to participate in the feasibility study. Patients will be asked to complete these questionnaires immediately following confirmation of their decision on participating in the study. Where this is not possible a copy of the questionnaire will be sent in the post by the local research team. Responses to these questionnaires will be confidential and patients will be identified only by their screening ID.

For peer review only - http://bmjopen.bmj.com/site/about/guidelines.xhtml

The results of this questionnaire will be analysed as an ongoing process to help inform and develop the approach of further patients.

Figure 1 shows the flow of participants through the trial.

Allocation and Blinding

Patients will be randomly allocated to the treatments on a 1:1 basis using a web-based randomisation procedure hosted by Bristol Randomised Trials Collaboration (a registered CTU) with concealment prior to consent, but no blinding of participants or clinical staff to the allocation of treatment pathway. The trial statistician is responsible for producing the allocation sequence, stratified by recruiting centre and minimised on Injury Severity Score (ISS) as an indicator of multiple injuries (<16 or >=16).

Interventions

Surgical management

Surgical management will involve fixation of the pelvic fracture by a specialist pelvic surgeon at the earliest opportunity. As surgical fixation of these fractures is performed regularly in all participating centres the method of fixation and choice of implant will be left to the operating surgeon. Post-operative management and rehabilitation will be left to the discretion of the treating surgeon. Details on the surgery and subsequent rehabilitation will be collected as part of the study.

Non-surgical management

Non-surgical management will be left to the discretion of the treating surgeon. Any decision on restricted weight-bearing will be left to the treating surgeon. Rehabilitation including Physiotherapy and Occupational Therapy will follow usual practices. Details on the rehabilitation will be collected as part of the study.

Outcomes

To assess the feasibility of the study design we will assess participant numbers such as recruitment rate, including numbers of patients meeting inclusion criteria and reasons for exclusion or declining where appropriate Compliance rates with allocated treatment and any reasons for not being able to comply. We will also look at follow-up rates, withdrawals, including reasons for withdrawal where appropriate in accordance with the CONSORT diagram. We will look at the outcomes measures that are expected to be used in the full trial

For peer review only

For peer review only - http://bmjopen.bmj.com/site/about/guidelines.xhtml

with particular interest in data completion rates, evidence of sensitivity to change (whether the score change over time) and whether the outcomes have ceiling or floor effects.

The following patient reported outcomes will be tested for use in a definitive study:

Measures at baseline and follow-up

Iowa pelvic score - A measure specific to outcomes following pelvic injury (Templeman et al[12]). Shows good construct validity when compared to the physical component of the SF-36. This is also the preferred pelvic specific outcome measure by patients[13] and the study patient advisory group.

Oxford Hip score (Dawson et al[14]) – A functional score for patients following hip injury and/or surgery. Whilst not pelvic specific the activities and symptoms included were felt to be relevant by our patient group.

EQ-5D-5L (Herdman et al[15]) - A standardised instrument of health status.

ICECAP-A (Al-Janabi et al[16]) - A measure of capability for the general adult population for use in economic evaluation. It focuses on wellbeing in the broader sense, not just health status.

Brief Pain Inventory (Cleeland[17]) - Originally developed to measure pain in patients suffering from cancer. It has since been used in a variety of conditions. It allows patients to rate the severity of their pain as well as its influence on their psychological health and activity

All participants will complete these questionnaires at baseline, 2 and 6 weeks, 3 and 6 months following randomisation. Participants recruited in the first 12 months of the study will also complete questionnaires at 9 and 12 months following randomisation. Baseline data will be collected at recruitment. Participants will be able to complete their follow-up questionnaires in person, when attending an outpatient appointment, online or by post. Standard care for participants with these injuries would be for clinical review in an outpatient clinic at 6 weeks, 3 months and 12 months (see table 1).

At these time points, in addition to the questionnaires, participants will complete a Timed Up and Go assessment (Podsiadlo & Richardson[18]). This is an assessment of a participant's physical walking ability and involves being timed to stand from a chair, walk a distance of 3m and return to sit in the chair. Where possible this will be completed by an assessor blinded to the participant's treatment allocation. Completeness of this assessment will be recorded to inform the appropriateness of its use in a definitive trial.

For peer review only - http://bmjopen.bmj.com/site/about/guidelines.xhtml

Data obtained as part of the study will be entered on to a secure password protected online REDCap database.

Study Duration

Recruitment will continue for 18 months. Follow-up for 6 months with 6 months for analysis.

	Baseline	2 weeks* (+ 1 week)	6 weeks* (+/- 1 week)	3 months* (+/- 2 weeks)	6 months* (+/- 3 weeks)	9 months* (+/- 3 weeks)	12 months* (+/- 4 weeks)
	Inpatient	*Phone/online*	*Clinic*	*Clinic*	*Post/online*	*Post/online*	*Clinic*
**Demographics**	✓						
**Injury characteristics**	✓						
**Clinical review**	✓	✓ [b]	✓	✓			✓
**Surgical details**		✓ [c]					
**Rehabilitation**		✓	✓	✓	✓	✓	✓
**Adverse Events**	✓	✓	✓	✓	✓	✓	✓
**Iowa Pelvic score**	✓ [a]	✓	✓	✓	✓	✓	✓
**OHS**	✓ [a]	✓	✓	✓	✓	✓	✓
**EQ-5D-5L**	✓ [a]	✓	✓	✓	✓	✓	✓
**ICECAP-A**	✓	✓	✓	✓	✓	✓	✓
**BPI**	✓ [a]	✓	✓	✓	✓	✓	✓
**TUAG**			✓	✓			✓
**Resource Use**			✓	✓	✓	✓	✓

[a]pre- & post-injury, [b]non-operative group only, [c]surgical group only, *from date of randomisation

Table 1 - Visit schedule

Economic Evaluation

The economic feasibility will focus on data collection to inform the economic evaluation to be done alongside the definitive trial. As well as the EQ-5D-5L and ICECAP-A we will record length of stay in both study arms, along with time spent in theatre and implants used (surgical arm only). Use of specific primary, community and social care services will be assessed by patient reported resource use questionnaires at 6 weeks, 3, 6, 9 and 12 months.

For peer review only

For peer review only - http://bmjopen.bmj.com/site/about/guidelines.xhtml

Qualitative Study

To inform the conduct of the definitive trial, we will invite up to 20 consented participants (10 from each treatment arm and across all sites) to take part in a semi-structured telephone interview by the qualitative researcher after they have completed the 6 month follow-up questionnaire. The interviews will explore their experience of the trial, their treatment and recovery, and acceptability of the outcome measures. A purposive sample will be selected to reflect maximum variation in socio-demographics, age and ethnicity. Topic guides for the interviews will be developed from the literature, team discussions and input from the PAG. Ten participating health care professionals (surgeons, research nurses and clinical nurse specialists) will be invited to take part in a telephone interview evaluating their experiences of treatment and views of trial processes.

Safety Reporting

Only serious adverse events will be reported for this study comparing two treatments in common clinical practice. A serious adverse event is any untoward medical occurrence that:

- results in death
- is life-threatening
- requires inpatient hospitalisation or prolongation of existing hospitalisation
- results in persistent or significant disability/incapacity
- consists of a congenital anomaly or birth defect

Serious adverse events which are expected with these injuries are:

- Wound complications/infections
- Neurovascular injury
- Thromboembolic events
- Chest infection
- Metal work/implant failure/loosening and non/mal-union

Secondary operations to prevent infection, mal-union, non-union or for symptoms related to the metalwork may also be expected.

Any unexpected serious adverse events will be recorded and reported to the Sponsor and Ethics Committee.

For peer review only

Sample Size

This feasibility study is designed to produce estimates of the parameters required to plan a definitive trial, together with enough data on outcome measures to show whether or not the ceiling effect on the Iowa instrument is likely to be a problem in the definitive trial. If 120 patients are screened as eligible and 40% agree to take part, then this will allow us to estimate the recruitment rate of 40% with a 95% confidence interval of 31% to 49% which is within 10% in either direction. Forty complete sets of data should be enough to show when a ceiling effect starts to occur although this will rely on a visual inspection of the data at each time point. If 60 sets of data are collected this will allow greater precision.

Data Analyses

Quantitative data analysis

As this is a feasibility trial no formal statistical testing will be carried out. Instead the analysis will focus on reporting data that will be used for planning and for assessing the feasibility of the definitive trial.

Feasibility parameters with 95% confidence intervals will be provided using the exact binomial method. The spread of the data and ceiling effects will be documented for all outcome variables using histograms for single time points and box plots to compare over time. Calculation of the area under the curve over time is the likely primary method of analysis for the definitive trial, and the feasibility analysis will investigate whether this would produce a sufficiently complete data set or whether it would be better to focus on a particular time point. The 95% confidence interval for the effect sizes for all potential outcome measures will be calculated to ensure that a future trial can be planned appropriately.

The future economic evaluation is likely to present results in cost/QALY terms reporting within trial and lifetime horizons. The economic feasibility work will focus on establishing the appropriate methods for collecting the outcomes, both costs and utilities, which will be of interest in the future economic evaluation, with analysis therefore limited to assessment of completeness and descriptive statistics.

Qualitative data analysis

With informed consent, all interviews will be digitally recorded, transcribed, anonymised and analysed using thematic methods of building codes into themes and sub-themes using the process of constant comparison (facilitated by NVIVO software: QSR International Pty Ltd).

For peer review only - http://bmjopen.bmj.com/site/about/guidelines.xhtml

This aspect is important to understand the acceptability of trial processes, including randomisation, treatment pathways and other outcome questionnaires for the definitive trial.

Patient and Public Involvement / Patient Advisory Group (PAG)

A patient advisory group (PAG) has been involved in the development of the study and advising on study design. The PAG have been particularly involved in the selection of appropriate outcome measures and reviewing patient facing materials including the information sheets. The group will continue to provide advice throughout the study and their advice on any changes which may improve recruitment and the study will be actively sought. A representative of the group will sit on the TSC to feedback the advice of the group to the committee. The PAG will also be actively involved in any publication and dissemination of results at the end of the study.

ETHICS AND DISSEMINATION

This manuscript is based on Protocol V.4.0 dated 08.05.2019. The study received South West – Central Bristol Research Ethics Committee (REC) approval on 2nd July 2018 and Health Research Authority approval on 4th July 2018. The trial will be conducted in accordance with the protocol, the principles of the Declaration of Helsinki and ICH GCP. Any amendments of the protocol will be submitted to the REC for approval. On request, the study investigators and their institutions will permit trial-related monitoring and audits by the Sponsor and relevant Research Ethics Committee. North Bristol NHS Trust is the nominated sponsor for this study.

A Trial Steering Committee (TSC) has been convened to provide overall supervision of the trial and ensure it is in accordance with the principles of good clinical practice and relevant regulations. The TSC agreed the trial protocol and will agree any protocol amendments. The TSC also provide advice to the investigators on all aspects of the trial including aspects of safety and monitoring of serious adverse events.

Dissemination

The findings of the study will be presented locally at each participating site and to the general orthopaedic community at national orthopaedic conferences. The findings will also

For peer review only - http://bmjopen.bmj.com/site/about/guidelines.xhtml

be submitted for publication in an open access peer-reviewed journal and presented at relevant conferences and research meetings.

For peer review only

For peer review only - http://bmjopen.bmj.com/site/about/guidelines.xhtml

REFERENCES

1. Burgess, A., Eastridge, B., Young, J., Ellison, T., Ellison, P., Poka, A., Bathon, G., Brumback, R. (1990) Pelvic ring disruptions: effective classification system and treatment protocols. *Journal of Trauma,* 30(7), pp848–856.
2. Manson, T., O'Toole, R., Whitney, A., Duggan, B., Sciadini, M., Nascone, J. (2010) Young-Burgess classification of pelvic ring fractures: does it predict mortality, transfusion requirements, and non-orthopaedic injuries? *Journal of Orthopaedic Trauma,* 24(10), pp603–609.
3. LeFaivre, K., Padalecki, J., Starr, A. (2009) What constitutes a Young and Burgess lateral compression-I (OTA 61-B2) pelvic ring disruption? A description of computed tomography-based fracture anatomy and associated injuries. *Journal of Orthopaedic Trauma,* 23, pp16–21.
4. Weaver, M., Bruinsma, W., Toney, E., Dafford, E., Vrahas, M. (2012) What are the patterns of injury and displacement seen in lateral compression pelvic fractures? *Clinical Orthopaedics and Related Research,* 470(8), pp2104-2110.
5. Bruce, B., Reilly, M., Sims, S. (2011) OTA Highlight paper predicting future displacement of nonoperatively managed Lateral Compression Sacral fractures: Can it be done? *Journal of Orthopaedic Trauma,* 25(9), pp523-527.
6. Sagi, H., Coniglione, F., Stanford, J. (2011) Examination under anesthetic for occult pelvic ring instability. *Journal of Orthopaedic Trauma,* 25, pp529–536.
7. Tosounidis, T., Kanakaris, N., Nikolaou, V., Tan, B., Giannoudis, P (2012) Assessment of lateral compression type 1 pelvic ring injuries by intraoperative manipulation: which fracture pattern is unstable? *International Orthopaedics,* 36, pp2553-2558.
8. Aprato, A., Joeris, A., Tosto, F., Kalampoki, V., Stucchi, A., Masse, A. (2016) Direct and indirect costs of surgically treated pelvic fractures. *Archives of Orthopaedic Trauma and Surgery,* 136, pp325-330.
9. Hoffmann, M., Jones, C., Sietsma, D. (2012) Persistent impairment after surgically treated lateral compression pelvic injury. *Clinical Orthopaedics and Related Research,* 470, pp2161-2172.
10. Hagen, J., Castillo, R., Dubina, A., Gaski, G., Manson, T. & O'Toole, R. (2016) Does surgical stabilization of lateral compression-type pelvic ring fractures decrease patients' pain, reduce narcotic use and improve mobilization? *Clinical Orthopaedics and Related Research,* 474(6), pp1422-1429.
11. Beckmann, J., Presson, A., Curtis, S., Haller, J., Stuart, A., Higgins, T., Kubiak, E. (2014) Operative agreement on lateral compression-1 pelvis fractures: A survey of 111 OTA members. *Journal of Orthopaedic Trauma,* 28(12), pp681-685.

For peer review only - http://bmjopen.bmj.com/site/about/guidelines.xhtml

12. Templeman, D., Goulet, J., Duwelius, P., Olson, S., Davidson, M. (1996) Internal fixation of displaced fractures of the sacrum. *Clinical Orthopaedics and Related Research,* 329, pp180-185.
13. LeFaivre, K., Slobogean, G., Ngai, J., Broekhuyse, H., O'Brien, P. (2014) What outcomes are important for patients after pelvic trauma? Subjective responses and psychometric analysis of three published pelvic-specific outcome instruments. *Journal of Orthopaedic Trauma,* 28(1), pp23-27.
14. Dawson, J., Fitzpatrick, R., Carr, A., Murray, D. (1996) Questionnaire on the perceptions of patients about total hip replacement. *Journal of Bone and Joint Surgery,* 78-B(2), pp185-190.
15. Herdman, M., Gudex, C., Lloyd, A., Janssen, M., Kind, P., Parkin, D., Bonsel, G., Badia, X. (2011) Development and preliminary testing of the new five-level version of EQ-5D (EQ-5D-5L) *Quality of Life Research,* 20(10), pp1727-1736.
16. Al-Janabi H, Flynn T, Coast J. (2012) Development of a self-report measure of capability wellbeing for adults: the ICECAP-A. *Quality of Life Research,* 21, pp167-176
17. Cleeland, C. (1989) Measurement of pain by subjective report. In: Chapman, C., Loeser, J. (eds) Advances in Pain Research and Therapy, Volume 12: Issues in Pain Measurement. New York: Raven Press, pp391-403.
18. Podsiadlo, D., Richardson, S. (1991) The timed "Up & Go": a test of basic functional mobility for frail elderly persons. *Journal of the American Geriatric Society,* 39(2), pp142-148.

For peer review only

Acknowledgements The authors would like to thank the patient representatives who contribute to the trial management and steering groups.

Author Contributions:

Steven Barnfield drafted the manuscript.

Jenny Ingram contributed to revisions to the manuscript and approved the final version

Ruth Halliday contributed to revisions to the manuscript and approved the final version

Xavier Griffin contributed to revisions to the manuscript and approved the final version

Rosemary Greenwood contributed to revisions to the manuscript and approved the final version

Rebecca Kandiyali contributed to revisions to the manuscript and approved the final version

Julian Thompson contributed to revisions to the manuscript and approved the final version

Joel Glynn contributed to revisions to the manuscript and approved the final version

Lucy Beasant contributed to revisions to the manuscript and approved the final version

John McArthur contributed to revisions to the manuscript and approved the final version

Peter Bates contributed to revisions to the manuscript and approved the final version

Mehool Acharya conceived the study, has contributed to revisions to the manuscript and approved the final version.

Funding: This work is funded by the National Institute for Health Research (NIHR) Research for Patient Benefit research programme grant number PB-PG-0816-20013.

This work is supported by the National Institute for Health Research Oxford Biomedical Research Centre

Disclaimer: This paper presents independent research funded by the National Institute for Health Research (NIHR) under its Research for Patient Benefit (RfPB) Programme (Grant Reference Number PB-PG-0816-20013). The views expressed are those of the author(s) and not necessarily those of the NIHR or the Department of Health and Social Care.

Competing interests: Xavier Griffin is funded by a National Institute for Health Research Clinician Scientist Grant. Further funding from industry and charitable grants are and have been made available to his institution. All decisions relating to the design, conduct, analysis,

For peer review only - http://bmjopen.bmj.com/site/about/guidelines.xhtml

write-up and publication of this research are independent of these funders. He has ongoing expert consultancy with several companies; none involve the development of any implant for use in pelvic fracture care.

No other authors have competing interests to declare.

Provenance and peer review: Not commissioned; externally peer reviewed.

Data sharing statement: The results of this feasibility study will inform the planning of a definitive randomised controlled trial, including whether it can be conducted, by assessing rates of recruitment, retention and data collection. Data will be made available upon reasonable request.

Trial status: Ongoing data collection.

Trial sponsor: North Bristol NHS Trust, Bristol BS10 5NB: researchsponsor@nbt.nhs.uk

Figure 1; Study flow diagram

For peer review only

For peer review only - http://bmjopen.bmj.com/site/about/guidelines.xhtml

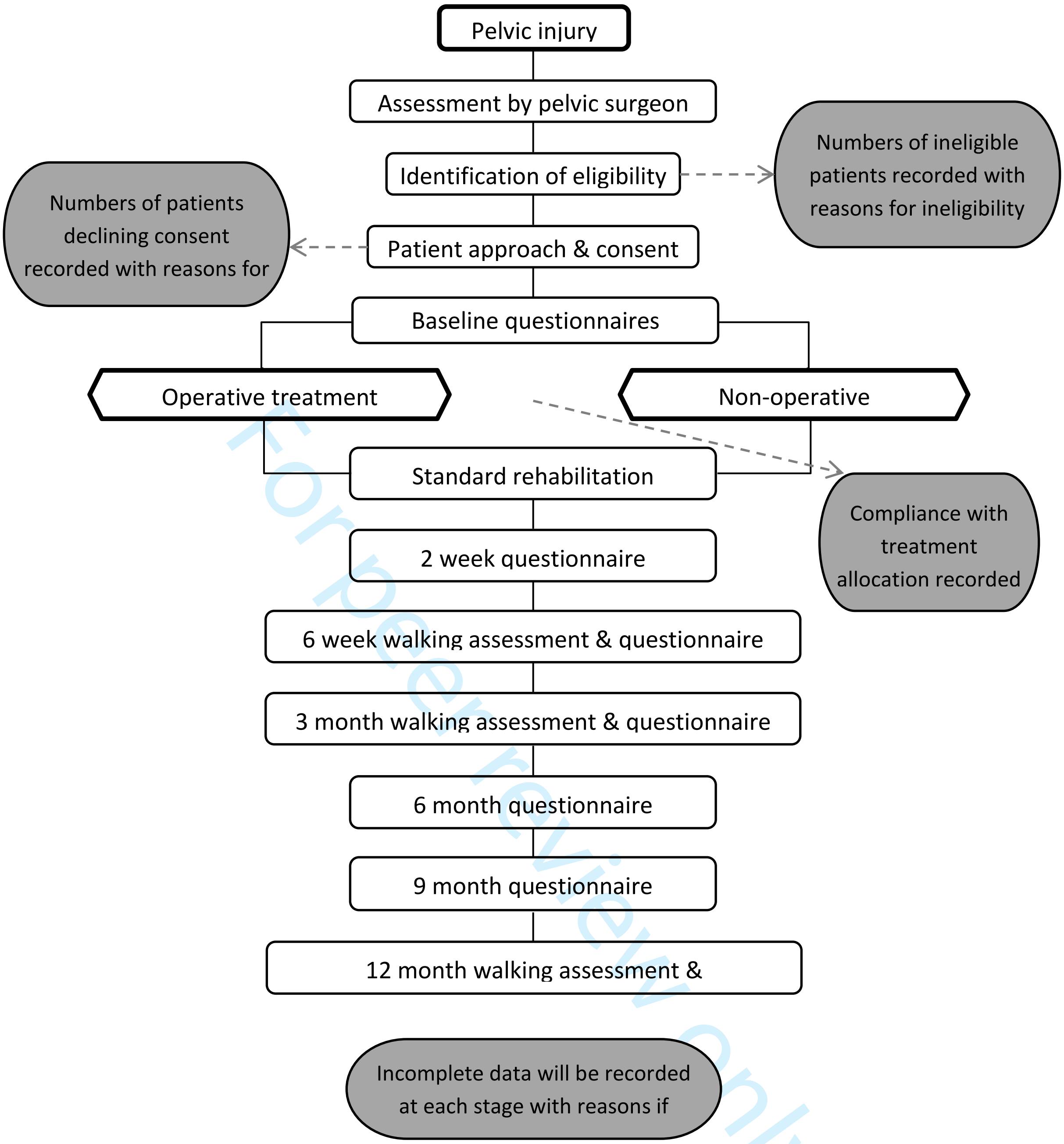

Figure 1 Study flow diagram

SPIRIT
STANDARD PROTOCOL ITEMS: RECOMMENDATIONS FOR INTERVENTIONAL TRIALS

SPIRIT 2013 Checklist: Recommended items to address in a clinical trial protocol and related documents*

Section/item	Item No	Description	Addressed on page number
**Administrative information**			
Title	1	Descriptive title identifying the study design, population, interventions, and, if applicable, trial acronym	_____1_______
Trial registration	2a	Trial identifier and registry name. If not yet registered, name of intended registry	_____3_______
	2b	All items from the World Health Organization Trial Registration Data Set	_____n/a_____
Protocol version	3	Date and version identifier	_____13_______
Funding	4	Sources and types of financial, material, and other support	_____17_______
Roles and responsibilities	5a	Names, affiliations, and roles of protocol contributors	_____1________
	5b	Name and contact information for the trial sponsor	_____17_______
	5c	Role of study sponsor and funders, if any, in study design; collection, management, analysis, and interpretation of data; writing of the report; and the decision to submit the report for publication, including whether they will have ultimate authority over any of these activities	_____n/a______
	5d	Composition, roles, and responsibilities of the coordinating centre, steering committee, endpoint adjudication committee, data management team, and other individuals or groups overseeing the trial, if applicable (see Item 21a for data monitoring committee)	_______n/a_____

For peer review only

**Introduction**			
Background and rationale	6a	Description of research question and justification for undertaking the trial, including summary of relevant studies (published and unpublished) examining benefits and harms for each intervention	____4_______
	6b	Explanation for choice of comparators	_____6______
Objectives	7	Specific objectives or hypotheses	_____6______
Trial design	8	Description of trial design including type of trial (eg, parallel group, crossover, factorial, single group), allocation ratio, and framework (eg, superiority, equivalence, noninferiority, exploratory)	_______6______
**Methods: Participants, interventions, and outcomes**			
Study setting	9	Description of study settings (eg, community clinic, academic hospital) and list of countries where data will be collected. Reference to where list of study sites can be obtained	_____7______
Eligibility criteria	10	Inclusion and exclusion criteria for participants. If applicable, eligibility criteria for study centres and individuals who will perform the interventions (eg, surgeons, psychotherapists)	_____7_______
Interventions	11a	Interventions for each group with sufficient detail to allow replication, including how and when they will be administered	_____8______
	11b	Criteria for discontinuing or modifying allocated interventions for a given trial participant (eg, drug dose change in response to harms, participant request, or improving/worsening disease)	_____n/a_____
	11c	Strategies to improve adherence to intervention protocols, and any procedures for monitoring adherence (eg, drug tablet return, laboratory tests)	_____n/a_____
	11d	Relevant concomitant care and interventions that are permitted or prohibited during the trial	_____8______
Outcomes	12	Primary, secondary, and other outcomes, including the specific measurement variable (eg, systolic blood pressure), analysis metric (eg, change from baseline, final value, time to event), method of aggregation (eg, median, proportion), and time point for each outcome. Explanation of the clinical relevance of chosen efficacy and harm outcomes is strongly recommended	_____9______
Participant timeline	13	Time schedule of enrolment, interventions (including any run-ins and washouts), assessments, and visits for participants. A schematic diagram is highly recommended (see Figure)	_____10_____

For peer review only - http://bmjopen.bmj.com/site/about/guidelines.xhtml

Sample size	14	Estimated number of participants needed to achieve study objectives and how it was determined, including clinical and statistical assumptions supporting any sample size calculations	_____12_____
Recruitment	15	Strategies for achieving adequate participant enrolment to reach target sample size	_____7______

Methods: Assignment of interventions (for controlled trials)

Allocation:

Sequence generation	16a	Method of generating the allocation sequence (eg, computer-generated random numbers), and list of any factors for stratification. To reduce predictability of a random sequence, details of any planned restriction (eg, blocking) should be provided in a separate document that is unavailable to those who enrol participants or assign interventions	______8______
Allocation concealment mechanism	16b	Mechanism of implementing the allocation sequence (eg, central telephone; sequentially numbered, opaque, sealed envelopes), describing any steps to conceal the sequence until interventions are assigned	______8______
Implementation	16c	Who will generate the allocation sequence, who will enrol participants, and who will assign participants to interventions	______8______
Blinding (masking)	17a	Who will be blinded after assignment to interventions (eg, trial participants, care providers, outcome assessors, data analysts), and how	______8_____
	17b	If blinded, circumstances under which unblinding is permissible, and procedure for revealing a participant's allocated intervention during the trial	_____n/a_____

Methods: Data collection, management, and analysis

Data collection methods	18a	Plans for assessment and collection of outcome, baseline, and other trial data, including any related processes to promote data quality (eg, duplicate measurements, training of assessors) and a description of study instruments (eg, questionnaires, laboratory tests) along with their reliability and validity, if known. Reference to where data collection forms can be found, if not in the protocol	_____9______
	18b	Plans to promote participant retention and complete follow-up, including list of any outcome data to be collected for participants who discontinue or deviate from intervention protocols	______n/a______

For peer review only - http://bmjopen.bmj.com/site/about/guidelines.xhtml

Data management	19	Plans for data entry, coding, security, and storage, including any related processes to promote data quality (eg, double data entry; range checks for data values). Reference to where details of data management procedures can be found, if not in the protocol	______10______
Statistical methods	20a	Statistical methods for analysing primary and secondary outcomes. Reference to where other details of the statistical analysis plan can be found, if not in the protocol	______12______
	20b	Methods for any additional analyses (eg, subgroup and adjusted analyses)	______12______
	20c	Definition of analysis population relating to protocol non-adherence (eg, as randomised analysis), and any statistical methods to handle missing data (eg, multiple imputation)	______n/a_____
**Methods: Monitoring**			
Data monitoring	21a	Composition of data monitoring committee (DMC); summary of its role and reporting structure; statement of whether it is independent from the sponsor and competing interests; and reference to where further details about its charter can be found, if not in the protocol. Alternatively, an explanation of why a DMC is not needed	______n/a______
	21b	Description of any interim analyses and stopping guidelines, including who will have access to these interim results and make the final decision to terminate the trial	_____n/a_____
Harms	22	Plans for collecting, assessing, reporting, and managing solicited and spontaneously reported adverse events and other unintended effects of trial interventions or trial conduct	_____11______
Auditing	23	Frequency and procedures for auditing trial conduct, if any, and whether the process will be independent from investigators and the sponsor	_____n/a_____
**Ethics and dissemination**			
Research ethics approval	24	Plans for seeking research ethics committee/institutional review board (REC/IRB) approval	_____13_____
Protocol amendments	25	Plans for communicating important protocol modifications (eg, changes to eligibility criteria, outcomes, analyses) to relevant parties (eg, investigators, REC/IRBs, trial participants, trial registries, journals, regulators)	_____13_____

For peer review only - http://bmjopen.bmj.com/site/about/guidelines.xhtml

Consent or assent	26a	Who will obtain informed consent or assent from potential trial participants or authorised surrogates, and how (see Item 32)	_____7______
	26b	Additional consent provisions for collection and use of participant data and biological specimens in ancillary studies, if applicable	____n/a______
Confidentiality	27	How personal information about potential and enrolled participants will be collected, shared, and maintained in order to protect confidentiality before, during, and after the trial	_____n/a_____
Declaration of interests	28	Financial and other competing interests for principal investigators for the overall trial and each study site	_____17______
Access to data	29	Statement of who will have access to the final trial dataset, and disclosure of contractual agreements that limit such access for investigators	_____n/a_____
Ancillary and post-trial care	30	Provisions, if any, for ancillary and post-trial care, and for compensation to those who suffer harm from trial participation	_____n/a_____
Dissemination policy	31a	Plans for investigators and sponsor to communicate trial results to participants, healthcare professionals, the public, and other relevant groups (eg, via publication, reporting in results databases, or other data sharing arrangements), including any publication restrictions	_____13______
	31b	Authorship eligibility guidelines and any intended use of professional writers	_____n/a______
	31c	Plans, if any, for granting public access to the full protocol, participant-level dataset, and statistical code	_____n/a______
**Appendices**			
Informed consent materials	32	Model consent form and other related documentation given to participants and authorised surrogates	_____n/a______
Biological specimens	33	Plans for collection, laboratory evaluation, and storage of biological specimens for genetic or molecular analysis in the current trial and for future use in ancillary studies, if applicable	_____n/a______

*It is strongly recommended that this checklist be read in conjunction with the SPIRIT 2013 Explanation & Elaboration for important clarification on the items. Amendments to the protocol should be tracked and dated. The SPIRIT checklist is copyrighted by the SPIRIT Group under the Creative Commons "Attribution-NonCommercial-NoDerivs 3.0 Unported" license.

For peer review only - http://bmjopen.bmj.com/site/about/guidelines.xhtml
