# Supplementary material for: TULIP: a randomised controlled trial of surgical versus non-surgical treatment of lateral compression injuries of the pelvis with complete sacral fractures (LC1) in the non-fragility fracture patient—a feasibility study protocol
Source: BMJ Open. 2020 Feb 10;10(2):e036588. doi: 10.1136/bmjopen-2019-036588 (PMC7044852; doi:10.1136/bmjopen-2019-036588)
Supplement: Reviewer comments [file bmjopen-2019-036588.reviewer_comments.pdf]

PEER REVIEW HISTORY

BMJ Open publishes all reviews undertaken for accepted manuscripts. Reviewers are asked to complete a checklist review form (**http://bmjopen.bmj.com/site/about/resources/checklist.pdf**) and are provided with free text boxes to elaborate on their assessment. These free text comments are reproduced below.

ARTICLE DETAILS

**TITLE (PROVISIONAL)**	TULIP: A Randomised Controlled Trial of Surgical versus Non-Surgical Treatment of Lateral Compression Injuries of the Pelvis with Complete Sacral Fractures (LC1) in the Non-fragility Fracture Patient - A Feasibility Study Protocol.
**AUTHORS**	Barnfield, Steven; Ingram, Jenny; Halliday, Ruth; Griffin, Xavier; Greenwood, Rosemary; Kandiyali, Rebecca; Thompson, Julian; Glynn, Joel; Beasant, Lucy; McArthur, John; Bates, Peter; Acharya, Mehool

This article was not externally reviewed at BMJ Open. Protocols that have been independently assessed prior to submission to BMJ Open are usually fast-tracked to publication on the grounds that further substantial changes will not be possible. This independent assessment will usually be external, independent review for both a substantial grant award from a non-commercial or government funder and ethics approval.
